# Decoding Cyclodextrins in Skin Applications: Structure, Mechanisms, and Safety in Cosmetic and Dermatologic Formulations (Part I)

**DOI:** 10.3390/ijms27115028

**Published:** 2026-06-02

**Authors:** Daniela-Ioana Mitrofan-Bandol, Ema-Teodora Nițu, Luciana Buliga, Alex-Robert Jîjie, Oana Andrada Iftode, Amalia Ridichie, Adriana Ledeți, Ionuț Ledeți, Laura Sbârcea

**Affiliations:** 1 Doctoral School, “Victor Babeş” University of Medicine and Pharmacy, 2nd Eftimie Murgu Street, 300041 Timișoara, Romania; ioana.mitrofan@umft.ro (D.-I.M.-B.); ema-teodora.nitu@umft.ro (E.-T.N.); luciana.buliga@umft.ro (L.B.); 2 Advanced Instrumental Screening Center, Faculty of Pharmacy, “Victor Babeş” University of Medicine and Pharmacy, 2nd Eftimie Murgu Square, 300041 Timișoara, Romania; amalia.ridichie@umft.ro (A.R.); afulias@umft.ro (A.L.); ionut.ledeti@umft.ro (I.L.); sbarcea.laura@umft.ro (L.S.); 3 University Clinic of Toxicology, Drug Industry, Management and Legislation, and Dermatopharmacy, Faculty of Pharmacy, “Victor Babeș” University of Medicine and Pharmacy, 2nd Eftimie Murgu Street, 300041 Timișoara, Romania; 4 Research Centre for Pharmaco-Toxicological Evaluation, Faculty of Pharmacy, “Victor Babeș” University of Medicine and Pharmacy, 2nd Eftimie Murgu Street, 300041 Timișoara, Romania; 5 Department of Analytical Chemistry, Faculty of Pharmacy, “Victor Babeș” University of Medicine and Pharmacy, 2nd Eftimie Murgu Street, 300041 Timișoara, Romania; 6 Faculty of Industrial Chemistry and Environmental Engineering, University Politehnica Timisoara, 2nd Victoriei Square, 300006 Timișoara, Romania; 7 Department of Physical Chemistry, Faculty of Pharmacy, “Victor Babeş” University of Medicine and Pharmacy, 2nd Eftimie Murgu Street, 300041 Timișoara, Romania; 8 Department of Drug Analysis, Faculty of Pharmacy, “Victor Babeş” University of Medicine and Pharmacy, 2nd Eftimie Murgu Street, 300041 Timișoara, Romania

**Keywords:** cyclodextrins, molecular encapsulation, skin penetration, safety assessment, botanical extracts, regulatory framework, dermocosmetics, topical delivery

## Abstract

Cyclodextrins (CDs) have emerged as important encapsulation platforms in dermatologic and cosmetic sciences, addressing critical challenges related to the solubility and stability of bioactive compounds. This review (Part I) systematically explores the relationship between the molecular architecture of CDs and their functional performance at the skin interface. A comprehensive analysis of the physicochemical properties of natural and derivative cyclodextrins is presented, alongside an evaluation of their interaction mechanisms with the skin barrier and current regulatory frameworks. This study highlights how the hydrophobic cavity of CDs enables the successful encapsulation of botanical extracts, significantly enhancing their bioavailability and photostability. Furthermore, the mechanistic insights provided clarify how appropriately selected and formulated CDs can modulate skin penetration while maintaining acceptable epidermal barrier compatibility. A critical synthesis of safety data and toxicological profiles confirms the suitability of specific CD types for topical application, supported by an overview of the evolving European and international legislative landscapes. Understanding the molecular design and safety constraints of cyclodextrin-based systems is essential for developing next-generation dermocosmetics. This foundational analysis provides the necessary framework for the clinical and technological applications discussed in the subsequent part of this review.

## 1. Introduction

Topical and transdermal delivery systems aim to deposit active ingredients into or across the integumentary system. Yet the integument, particularly the stratum corneum (SC), constitutes the principal barrier that modulates percutaneous absorption and the distribution of water, lipids, and proteins, thereby strongly influencing the penetration, retention, and bioavailability of topically applied actives [1,2]. The SC’s highly organized lipid and protein domains determine intercellular diffusivity and are sensitive to hydration and formulation-induced perturbations. Label-free imaging and vibrational techniques (e.g., stimulated Raman scattering) have been used to trace water and to interrogate protein–lipid organization across the SC, underscoring the complexity of the skin barrier that formulators must address [1]. Many dermatologic and cosmetic actives, particularly botanicals and lipophilic small molecules such as carotenoids, isoflavonoids (e.g., genistein), and other plant-derived polyphenols, are intrinsically poorly water-soluble, chemically labile (prone to oxidation, photodegradation, or thermal decomposition), and often volatile or possess undesirable organoleptic properties. These physicochemical features limit their incorporation, stability, and effective delivery in aqueous- or skin-compatible vehicles [3,4]. Contemporary dermatologic and cosmetic practices seek to combine efficacy with safety and consumer-acceptable aesthetics (e.g., odor control and texture), increasing the formulation complexity required to reconcile stability, potency, and cutaneous tolerability [3,5].

First discovered in 1891 by the French pharmacist and chemist Antoine Villiers, cyclodextrins (CDs) are cyclic oligosaccharides produced from starch by cyclodextrin glycosyltransferase (CGTase). The most widely used and best-characterized native CDs are α-, β-, and γ-CDs, containing six, seven, and eight D-glucopyranose units, respectively; however, larger-ring CDs, including δ-CD and higher homologs composed of nine or more glucopyranose units, have also been described and represent an expanding area of cyclodextrin research. These native CDs present a truncated conical (toroidal) architecture with a relatively hydrophobic inner cavity and a hydrophilic external surface. This geometry underpins their ability to form non-covalent inclusion complexes (ICs) with suitably sized guest molecules [6]. Inclusion complexation with CDs alters guests’ apparent solubility, chemical stability, and thermal behavior (e.g., changes or suppression of melting and sublimation events), as observed across multiple spectroscopic and thermal analytical studies of botanical actives such as genistein and other hydrophobic guests [4,7]. By hosting lipophilic moieties within their hydrophobic cavities while remaining outwardly water-compatible, CDs and their derivatives can therefore enhance the apparent aqueous solubility and dissolution rate of poorly soluble actives, protect labile molecules from oxidation, photodegradation, or volatilization, and modulate release kinetics and local availability at the skin surface or within epidermal compartments. Those attributes are directly relevant to both cosmetic and dermatologic applications [3,8]. Importantly, CD-based approaches are diverse: native CDs, chemically modified CDs (e.g., hydroxypropyl β-CD), cross-linked CD polymers (e.g., nanosponges), CD–polymer hybrids, and CD-based supramolecular assemblies (including CD metal–organic frameworks and CD–hydrogel hybrids) provide a toolbox to tailor complexation strength, release profiles, and carrier architecture to specific formulation goals [9,10,11].

Experimental and formulation studies across dermatologic and cosmetic matrices demonstrate the multifaceted roles that CDs may play in topical delivery. Complexation can improve active solubility and biocompatibility (for example, galangin/β-CD complexes improved aqueous compatibility and in vitro biocompatibility in oncology models) and has been exploited to stabilize plant-derived extracts such as carotenoids and propolis actives for downstream formulation and commercialization [4,8,12]. CDs have been formulated into topical semi-solid systems (e.g., liquid crystalline gels, nanosponge dispersions, and thermoresponsive nanogels), where they act both as complexing agents and as structural components of the carrier matrix. Such constructs have been shown to enhance dermal encapsulation and, in some designs, to increase skin penetration of hydrophobic payloads when combined with appropriate cross-linkers and nanocarrier architectures [10,13]. Nevertheless, the relationship between complexation and percutaneous flux is not unidirectional: the fraction of the free drug (uncomplexed) available for partitioning into the skin governs net flux, so that high concentrations of the complexing agent (e.g., hydroxypropyl β-CD in some gel formulations) may reduce the free drug and thus diminish immediate permeation, whereas carrier designs that release the guest at the skin interface or that combine CDs with permeation-promoting architectures can enhance net delivery [14]. The diversity of CD formats, from simple ICs to cross-linked nanosponges and CD-functionalized nanogels, thus enables optimization for competing objectives such as enhanced stability, controlled release, and targeted epidermal retention [3,11].

The implementation of CD-based strategies for skin application requires an integrated assessment of safety, stability, and regulatory status. β-cyclodextrin and its derivatives are frequently used in topical research and development (in part because their cavity size is compatible with many cosmetic actives), while α-CD may be too small for some guests, and γ-CD is less used owing to cost and production constraints. Consequently, the choice of CD type and chemical modification is commonly dictated by guest size, desired complexation strength, and safety considerations [2,5]. Cross-linked CD polymers (e.g., nanosponges) and CD-based nanomaterials introduce additional variables: cross-linker chemistry, network density, and environmental pH can affect stability and release behavior. These features have been the subject of acute and repeated-dose toxicological evaluation in preclinical studies. Research highlights that some CD derivatives have established regulatory acceptance for drug delivery while underscoring the need for nanotoxicological characterization when CDs are formulated in novel particulate or polymeric forms intended for skin contact, given differences in systemic exposure potential, dermal irritation, and local tolerability [2,5,15]. The regulatory and safety landscape, therefore, demands case-by-case evaluation that couples physicochemical characterization, biocompatibility testing, and an awareness of manufacturing variables that influence both product performance and risk.

Technologically, CDs enable both straightforward ICs (often serving as solubility enhancers or stabilizers in aqueous vehicles) and more advanced carrier systems (supramolecular hydrogels, affinity-based depots, nanosponges, and CD metal–organic frameworks) that expand functionality to controlled or stimuli-responsive release, oxygen transport, and improved encapsulation of complex botanical matrices [16,17]. However, challenges remain in achieving durable carrier stability, scalable and sustainable production, and reproducible drug-loading and release characteristics. Recent advances in mechanochemical and solvent-reduced syntheses (e.g., twin-screw extrusion) and in rational cross-linker selection aim to address scale-up and environmental sustainability while preserving functionality [10,18]. Moreover, the binding affinity between a given guest and a particular CD scaffold varies widely, necessitating systematic screening and thermodynamic characterization during formulation development to predict complexation-driven effects on solubility and release [19]. Practical formulation design therefore reconciles the thermodynamics of complexation, the kinetics of guest release at the skin interface, the physical architecture of the carrier, and the regulatory/safety constraints pertinent to the intended cosmetic or therapeutic indication [14,16].

Overall, cyclodextrins constitute a versatile and expanding set of tools for addressing the principal challenges of dermatologic and cosmetic formulation, improving solubility and stability of labile or hydrophobic actives, enabling novel carrier architectures, and offering routes for controlled and affinity-mediated delivery. However, their effective deployment requires careful matching of CD type and format to the properties of the guest, rigorous evaluation of safety and stability, and attention to scalable and sustainable manufacturing routes [2,5,10,20,21].

Several previous reviews have addressed the use of CDs and CD-based ICs in bioactive compound encapsulation, topical/non-invasive treatments, and drug delivery applications, highlighting their ability to improve apparent solubility and stability, reduce volatility and undesirable organoleptic properties, and support the controlled release of active ingredients [3,5,21]. These contributions have been essential in establishing CDs as valuable formulation excipients in skin-related and bioactive delivery systems. However, the available literature remains fragmented between general formulation applications, pharmaceutical delivery aspects, isolated classes of active ingredients, and technological case studies. In this context, the novelty of the present review lies in its integrated structure–mechanism–safety perspective. Rather than focusing only on product categories or individual formulation examples, Part I connects CD molecular architecture and derivative selection with IC formation mechanisms, skin barrier interactions, penetration modulation, toxicological considerations, regulatory constraints, and the encapsulation of plant-derived bioactive compounds. This integrative framework is intended to support a more rational selection of CD type and formulation strategy for dermatologic and cosmetic applications and to establish the mechanistic and safety basis for the formulation-oriented discussion presented in Part II.

Accordingly, this two-part review synthesizes contemporary evidence on the use of CDs as advanced encapsulation platforms for dermatological and cosmetic applications. The present article (Part I) provides a comprehensive overview of CD chemistry, including their molecular architecture, structural types, derivatization, and the mechanisms governing IC formation. It further delivers a focused analysis of CD–skin interactions, examining the physicochemical determinants of dermal delivery and the effects of these macrocycles on skin barrier function. Given the clinical importance of biocompatibility, this part explores the toxicological landscape and safety assessment of topical exposure, followed by a detailed account of the European and international legislative frameworks. Finally, Part I offers a systematic survey of cyclodextrin-assisted encapsulation of plant extracts, highlighting the improvement of solubility, stability, and bioavailability of botanical active ingredients. These foundational aspects establish the framework for Part II, which addresses specific cosmetic and dermatologic formulations, technological challenges, and future innovations in skin-targeted delivery.

## 2. Cyclodextrins: General Aspects, Structure, Types, and Applications

### 2.1. Structure and Physicochemical Properties

**Figure 1:**
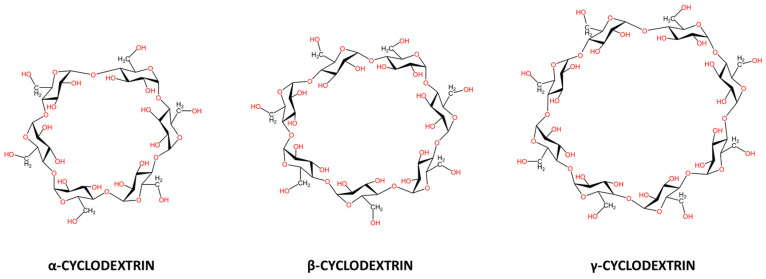
Chemical structures of the native cyclodextrins α-cyclodextrin, β-cyclodextrin, and γ-cyclodextrin, consisting of six, seven, and eight α-(1→4)-linked D-glucopyranose units, respectively, forming toroidal macrocycles with progressively increasing cavity sizes. The chemical structures were created using KingDrawHD v1.4.5.-20230617 software.

Cyclodextrins are derived from starch or its derivatives, acting as complexing agents that enhance the water solubility of compounds with poor aqueous solubility, thereby improving their solubility and bioavailability [22]. Cyclodextrins are cyclic oligosaccharides composed of α-(1→4)-linked D-glucopyranose units that adopt a truncated cone (torus) geometry with a relatively hydrophobic central cavity and a hydrophilic exterior bearing secondary and primary hydroxyl groups. This architecture underlies their host–guest inclusion chemistry. The three native CDs (α-, β-, and γ-cyclodextrin) contain six, seven, and eight glucopyranose units, respectively, and the progressive increase in ring size correlates with enlarged cavity volumes and altered guest size selectivity, a key determinant of complexation specificity (Figure 1). The spatial arrangement of the hydroxyl groups is stereochemically important: secondary hydroxyls at C2 and C3 form the wider (secondary) rim, whereas primary C6 hydroxyls define the narrower (primary) rim; this rim asymmetry and the pattern of intramolecular hydrogen bonds influence solubility, crystal packing, and reactivity [23,24].

The physicochemical behavior of native CDs is governed by the balance between cavity hydrophobicity and external hydrophilicity, by intra- and intermolecular hydrogen bonding, and by solid-state crystal lattice energies. For example, β-CD typically exhibits limited aqueous solubility due to stronger crystal packing and intramolecular hydrogen bonding compared with its hydroxypropylated or sulfobutylated derivatives [23,25,26]. Chemical modification of the rim hydroxyls (e.g., methylation, hydroxypropylation, and sulfobutylation) is routinely used to reduce crystallinity, disrupt unfavorable hydrogen bonding networks, and increase apparent aqueous solubility and formulation versatility, while also altering pharmacokinetic and toxicity profiles [25,26,27,28,29]. Complexation parameters (stoichiometry and binding constant K) and thermodynamic signatures (enthalpy versus entropy contributions) depend jointly on CD ring size and substitution pattern, guest molecular shape and polarity, and medium conditions (solvent, pH, and temperature). These determinants govern both the strength and the functional consequences of inclusion [23,24,28]. These modifications led to CDs with various applications in the local administration of drugs, such as heptakis (2,6-di-O-methyl)-β-cyclodextrin (dimethyl-β-cyclodextrin, DMβCD), randomly methylated-β-cyclodextrin (RMβCD), and heptakis (2,3,6-tri-O-methyl)-β-cyclodextrin (TMβCD), each containing seven glucose units; hydroxypropyl-α-cyclodextrin (HPαCD), hydroxypropyl-β-cyclodextrin (HPβCD), and hydroxypropyl-γ-cyclodextrin (HPγCD), containing six, seven, and eight glucose units, respectively; and sulfobutylether-β-cyclodextrin (SBEβCD), containing seven glucose units [30,31,32,33,34]. Larger cyclic oligosaccharides, generally referred to as larger-ring CDs, contain nine or more glucopyranose units and include δ-CD and higher homologs. Although they are less commonly used than α-, β-, and γ-CDs in current pharmaceutical and dermocosmetic applications, these larger-ring structures are gaining increasing attention because their expanded cavities may accommodate bulkier guests and enable distinct host–guest architectures [35].

The diameter of CDs increases with the number of glucose units in the order α-CD < β-CD < γ-CD. Their aqueous solubility at 25 °C also varies with the number of glucose units in the structure, with γ-CD being the most soluble (232.0 mg/mL), followed by α-CD (145.0 mg/mL) and β-CD (18.5 mg/mL) [30,36,37,38].

The physicochemical properties of cyclodextrins strongly influence their ability to form ICs and thus their suitability for pharmaceutical and medical applications. To highlight the differences between native structures and substituted derivatives, Table 1 summarizes the key characteristics of natural cyclodextrins (α-, β-, and γ-CD), including the number of glucose units, molecular weight, aqueous solubility, height, and outer and inner cavity diameters. Table 2 compiles the same parameters for a representative set of cyclodextrin derivatives (hydroxypropyl, methylated, and sulfobutylether CDs), while also indicating the average substitution degree or molar substitution associated with the reported molecular weight values. This distinction is important because the molecular masses of chemically modified CDs are not fixed constants, but average values that may vary according to substitution degree, substitution pattern, counterion form, and manufacturer specifications.

Because CDs are small oligosaccharides with defined cavities, their complexation properties and physicochemical signatures are amenable to a broad analytical toolkit: phase solubility profiling, isothermal titration calorimetry (ITC), 1H and 2D nuclear magnetic resonance (NMR), X-ray diffraction, differential scanning calorimetry/thermogravimetric analysis (DSC/TGA), infrared (IR) spectroscopy, and molecular modeling/docking are complementary methods commonly used to characterize host–guest stoichiometry, geometry, and energetics in research and product development [23,24,28].

### 2.2. Types of Cyclodextrins

The native (natural) cyclodextrin family (α, β, and γ) arises commercially from the enzymatic conversion of starch by cyclodextrin glycosyltransferase (CGTase). β-CD is the most frequently used native form in many applications because of production economics and a cavity size that accommodates a large fraction of small to moderately sized organic guests [23,24]. Regulatory and safety considerations have supported certain uses of CDs in food and consumer products. β-CD and other CDs have been included in generally recognized as safe (GRAS) evaluations and are deployed industrially for applications such as cholesterol removal and aroma stabilization [38,39].

A large palette of chemically substituted CDs has been developed to regulate solubility, complexation selectivity, toxicity, and in vivo disposition. Representative modifications include RMβCD, HPβCD, SBEβCD (Captisol^®^), and sulfoalkyl ether derivatives. These modifications decrease crystallinity, increase aqueous solubility, and can reduce cell or tissue incompatibility observed with certain unmodified forms in parenteral contexts [25,27,28]. SBEβCD (Captisol^®^) exemplifies a clinically relevant engineered derivative that enhances the solubility and stability of hydrophobic actives and has been profiled in both in vitro and formulation studies for reduced cytotoxicity compared with organic solvent vehicles [27,28]. Safety profiling at the preclinical level has focused on hemolysis, renal handling, and tissue reabsorption. Anionic sulfoalkyl derivatives were designed specifically to reduce parenteral reabsorption and thereby diminish systemic toxicity for injectable uses [25,40].

Beyond monomeric CDs, insoluble or extended CD architectures, cross-linked CD polymers, cyclodextrin-based metal–organic frameworks (CD-MOFs), and polysaccharide- or polymer-cross-linked CD complexes have been created to generate high-capacity sorbents, controlled-release matrices, and materials with tunable porosity and thermal stability [39,41]. Pectin-cross-linked β-CD constructs and other biopolymer–CD composites illustrate how cross-linking modifies thermal and release behavior while preserving inclusion capacity for sequestration or food/industrial processing applications [38,39]. CD-MOFs add crystallographically ordered, high-surface-area networks that extend CD host chemistry into adsorption, catalysis, and advanced drug delivery platforms [23,41].

Conjugation of CDs to targeting ligands or polymers permits active delivery strategies, such as folate-modified β-CDs and other ligand-conjugated derivatives investigated for cell-specific uptake in cancer or targeted delivery scenarios, thereby extending CD function beyond passive solubilization toward receptor-mediated targeting and payload delivery [23,42].

**Figure 2:**
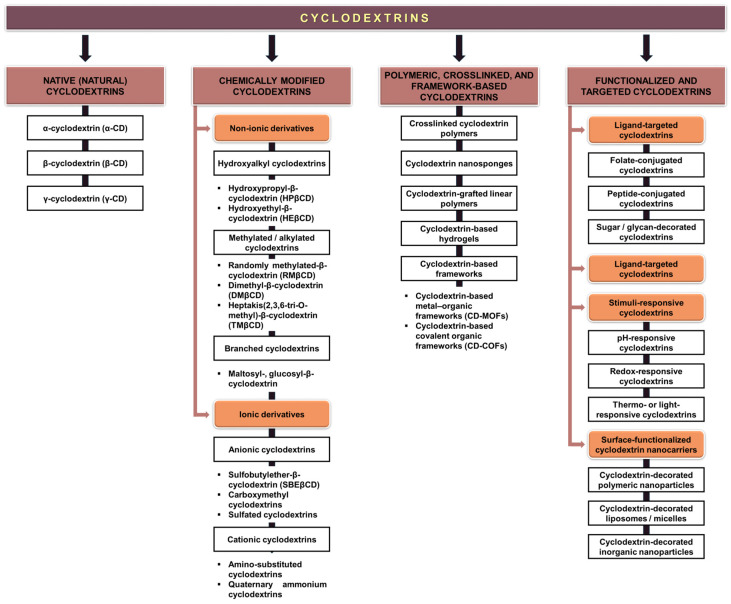
Schematic classification of cyclodextrins according to their level of structural modification, comprising native (natural) cyclodextrins, chemically modified cyclodextrins, and polymeric, cross-linked, and framework-based cyclodextrins, as well as functionalized and targeted cyclodextrins.

The main classes of cyclodextrins are summarized in Figure 2. Native (natural) cyclodextrins (α-, β-, and γ-cyclodextrins) serve as parent structures that can be further modified into chemically modified derivatives via non-ionic or ionic substitution. These building blocks can subsequently be assembled into polymeric, cross-linked, and framework-based cyclodextrins, and can also be engineered as functionalized and targeted cyclodextrins by attaching specific ligands or surface groups to endow them with tailored recognition, responsiveness, or delivery properties.

### 2.3. Inclusion Complex Formation and Mechanisms

IC formation between a CD and a guest molecule is principally non-covalent and results from the combined contribution of hydrophobic effects, van der Waals contacts, hydrogen bonding interactions at the cavity rims, and desolvation processes. In this context, the hydrophobic effect should be understood as arising from the release of structured, energetically unfavorable water molecules not only from the relatively apolar CD cavity but also from the hydrophobic surface of the guest molecule as host–guest contact is established. Guest inclusion, therefore, involves the simultaneous partial desolvation of both partners, followed by the replacement of water–host and water–guest contacts with direct host–guest interactions inside or near the CD cavity. The net free energy of complexation reflects the balance between these desolvation contributions, the favorable host–guest contact energies, possible hydrogen bonding at the rims, and any conformational adjustments of the guest or host induced by binding [23,24,26,28].

Although 1:1 host–guest complexes are most common, alternative stoichiometries (1:2, 2:1, and higher-order assemblies) occur when guest geometry, hydrophobic surface area, and rim substitution favor such packing arrangements. Observed association constants vary widely (from modest values to very strong complexes) depending on the CD derivative, guest chemistry, and solvent conditions [23,24,28,43,44,45]. Determination of binding constants and thermodynamic signatures is routinely accomplished by ITC, NMR titration, phase solubility analysis, and modeling to inform formulation choices [23,24,28].

Inclusion modifies multiple physicochemical attributes of guest molecules: aqueous solubility is frequently increased, photochemical or oxidative degradation is reduced, volatility and off odors can be masked, and dissolution rate and oral or topical bioavailability can be improved [23,26,27,28]. Representative, well-documented examples include the enhanced aqueous solubility of crystalline astaxanthin when SBEβCD (Captisol^®^) is used [27], the improved dissolution performance of glimepiride when complexed with Captisol^®^ [28], the increased solubility and oral bioavailability of flavonoids such as myricetin upon complexation [26], and the use of β-CD for cholesterol removal in food matrices through selective inclusion and complex precipitation [38].

**Figure 3:**
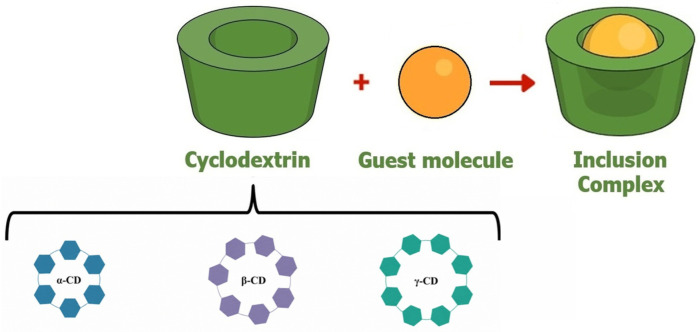
Schematic illustration of inclusion complex formation between a cyclodextrin host and a guest molecule. Native α-, β-, and γ-cyclodextrins, differing in cavity size, can entrap suitably sized guest molecules within their hydrophobic inner cavity to form non-covalent inclusion complexes.

Inclusion complexation is a key feature of cyclodextrin chemistry. As shown in Figure 3, the toroidal cyclodextrin host accommodates an appropriately sized guest molecule within its apolar cavity, driven mainly by hydrophobic interactions, van der Waals forces, and, in some cases, hydrogen bonding. The size and shape complementarity between the guest and the cavity of α-, β-, or γ-cyclodextrin largely determines the stability and stoichiometry of the resulting IC.

A robust experimental toolbox supports formulation and characterization of CD complexes: phase solubility diagrams and NMR proximity methods map binding stoichiometry and geometry; DSC, XRD, and FTIR probe solid-state changes; ITC yields direct thermodynamic data; and molecular docking complements experimental work to predict binding orientations and energetics. Common preparative routes for ICs include solution complexation followed by freeze-drying or spray-drying, co-evaporation, kneading, co-precipitation, and mechanochemical grinding. The chosen method influences residual solvent, crystallinity, and, ultimately, release and stability in the intended formulation matrix [23,26,28,46,47,48].

Among the available characterization methods, solution-state NMR spectroscopy, particularly ^1^H-NMR, DOSY, ROESY/NOESY, and related experiments performed in D_2_O when the guest–CD system is sufficiently soluble, provides especially valuable evidence for IC formation. Chemical shift variations of CD inner-cavity protons and guest resonances, diffusion coefficient changes, and through-space ROESY/NOESY cross-peaks can support host–guest proximity, complex stoichiometry, and the preferential orientation of the guest inside the CD cavity. Importantly, when spectra are acquired in D_2_O, the observation of guest resonances in aqueous solution may also indirectly support improved aqueous solubilization of poorly water-soluble actives. However, NMR data should be interpreted together with phase solubility analysis, dissolution testing, and complementary solid-state methods because extract complexity, signal overlap, limited guest solubility, and formulation matrix effects may restrict the applicability of D_2_O-NMR for some botanical systems [49,50,51].

### 2.4. General Applications Across Industries

In recent years, CDs have been increasingly employed in various pharmaceutical formulations, following their approval by several regulatory agencies [6,52,53,54].

Most applications of CDs are related to their ability to form ICs. Both CDs and their derivatives are frequently used as functional excipients to improve the solubility, permeability, stability, and bioavailability of active pharmaceutical ingredients (APIs), but also to counteract certain adverse effects, such as irritation, and to mask unpleasant odors. In pharmaceutical applications, CDs have the potential to influence the behavior and therapeutic efficacy of drugs. Clinical studies have revealed new applications for CDs, including CD-based nanoparticles, protein drug stabilization, and ready-to-use injection systems [23,35,52,53,54,55,56].

CDs are widely applied in drug formulation to solubilize poorly water-soluble APIs, stabilize labile molecules, enable parenteral administration of hydrophobic drugs (notably using modified derivatives such as SBEβCD/Captisol^®^), reduce local irritation, and modulate release kinetics in topical and systemic preparations [23,27,28,40]. In addition to classical oral and parenteral uses, CDs and selected derivatives have been investigated for pulmonary delivery (nebulization) strategies with attention to inhalation safety profiles for different derivatives and dose regimens [23,57].

**Figure 4:**
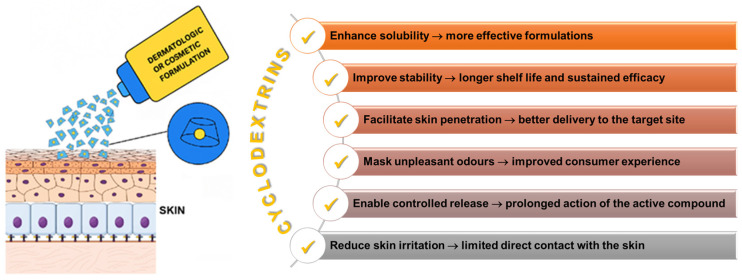
The main benefits of incorporating cyclodextrins into dermatologic or cosmetic formulations for topical application to the skin. The figure was created using Servier Medical Art (licensed under Creative Commons Attribution 3.0 Unported License).

In dermopharmaceutical and cosmetic formulations, the use of CDs has attracted attention since the 1970s due to their ability to enhance skin permeability and enable controlled delivery to the target site while maintaining skin biocompatibility. Nowadays, CDs are exploited to solubilize and protect botanical actives, antioxidants, and photolabile actives, reduce irritation potential by controlling free-active concentrations, and enhance aesthetic attributes (odor masking and reduced greasiness through inclusion) while improving storage stability (Figure 4). Comparative formulation studies (for example, ICs versus phytosomes for plant extracts) demonstrate that CD complexation can markedly increase apparent solubility and modulate toxicity and delivery profiles relevant to topical applications [23,24,26,56,58,59,60]. These attributes make CDs particularly attractive for the encapsulation of botanical ingredients and for cosmeceutical formulations that require both stability and acceptable skin tolerability [23,26,56,58,59,60].

CDs are tasteless, odorless, colorless, biocompatible, biodegradable, and non-carcinogenic compounds characterized by low toxicity and high stability [58]. Due to their versatility, these carriers of active ingredients show great potential both in the treatment of dermatological disorders (psoriasis, acne, dermatitis, microbial skin infections, wound healing, onychomycosis, and skin cancer) and in the development of advanced cosmetic formulations [60]. CDs have also demonstrated effectiveness as carriers of active ingredients in topical products such as sunscreens, antiperspirants, deodorants, and anti-aging formulations, as well as shampoos and fragrances, which are designed to act on the skin surface without systemic absorption [60,61].

CDs are also used in food science, where they form ICs with natural and plant-derived bioactive compounds, enhancing their stability and bioavailability [36]. The capacity of CDs to form selective ICs underpins industrial applications that include cholesterol removal from fats and dairy products (such as butter, milk, and eggs, achieving up to an 80% reduction in cholesterol content), stabilization and controlled release of flavors and aromas, and taste/odor masking. These applications are supported by GRAS evaluations and by scalable CD-based processing methods [23,38,39,62]. From a metabolic perspective, α- and β-CD are fermented by the intestinal microbiota and are resistant to enzymatic digestion, whereas γ-CD is almost completely hydrolyzed by α-amylase into glucose. Consequently, γ-CD exhibits intrinsic metabolic activity, opening new avenues for applications [63].

Extended CD constructs such as cross-linked polymers and CD-MOFs are applied in adsorption, separation, catalysis, and controlled-release systems that span environmental remediation, analytical separations, and materials science, showing the selectivity of the CD cavity within robust solid matrices [23,39,41]. Cross-linked and frameworked CDs expand the utility of inclusion chemistry into high-capacity, reusable sorbents and advanced delivery scaffolds [39,41]. CDs used in environmental processes aid in the removal of organic pollutants and heavy metals from water and soil through encapsulation and adsorption mechanisms and are incorporated into eco-friendly insecticide formulations. CDs are also applied in the textile industry as auxiliary agents that regulate dye release and improve color uniformity, and in packaging, where CD-based ICs are used in antimicrobial and volatile coatings to preserve the freshness and quality of products [36,64,65].

The modular chemistry of CDs supports functionalization for targeting ligands, integration into nanoscale carriers (for instance, nanosponges or polymeric CD networks), and conjugation into more complex architectures for controlled and targeted delivery. Such approaches are under active preclinical investigation to combine inclusion chemistry with receptor targeting, stimuli-responsive release, and material-driven sequestration strategies [23,40,41,42].

The structural simplicity yet chemical tunability of cyclodextrins make them versatile encapsulating agents that bridge solubilization, stabilization, and delivery needs across pharmaceuticals, foods, cosmetics, and materials science. However, the functional outcomes and safety profile are strongly dependent on the specific CD type, substitution pattern, and formulation process, which are aspects discussed in the subsequent chapters on skin interaction, safety assessment, and regulatory elements.

## 3. Interaction of Cyclodextrins with the Skin

### 3.1. Mechanisms of Skin Penetration Enhancement or Modulation

The literature supports two mechanisms by which CDs influence topical delivery: (i) indirect modulation of the free/unbound drug concentration, release rate, and partitioning behavior through guest inclusion and vehicle effects, and (ii) direct physicochemical interactions with SC lipids, producing structural perturbation of the barrier.

CDs used as encapsulating agents alter the apparent solubility, dissolution behavior, and release kinetics of lipophilic actives in the vehicle, thereby changing the fraction of unbound drug available to partition into the SC. Such modulation of free drug concentration and partitioning is a primary determinant of transcutaneous flux in established permeation models. Encapsulation does not uniformly increase flux: mathematical and experimental analyses highlight that enhanced aqueous solubilization of an active (for example, via IC formation) can either increase or decrease net skin uptake, depending on the guest- and derivative-dependent stability of the complex, the dissociation/release kinetics at the vehicle–skin interface, and the vehicle–SC partition coefficients that govern the driving force for diffusion [66,67]. Empirically, encapsulating strategies can increase skin deposition when they favor drug release within or immediately adjacent to the SC, indicating that the encapsulation–release balance is formulation-critical [68].

In addition to indirect effects on drug activity, CDs can interact directly with lipids of biological membranes. Preclinical work has documented the extraction of lipophilic skin constituents (including free fatty acids, phospholipids, and cholesterol) by cyclodextrins and cyclodextrin derivatives, and the interaction of native β-cyclodextrin surfaces with lipoprotein-like structures. It was observed that water-soluble substituted derivatives show different (often reduced) lipid-binding behavior relative to parent β-CD [69,70,71]. The SC barrier relies on highly ordered intercellular lipid lamellae (principally ceramides, cholesterol, and free fatty acids) for its impermeability, and the removal or binding of those lipids by an excipient will perturb lamellar order and increase permeability [72,73,74].

By preventing surface crystallization and accelerating the dissolution of poorly soluble ingredients, CDs can increase the chemical activity of the drug at the skin surface and therefore its driving force for penetration. This behavior has been documented in hydrogel and nanosponge contexts in which CD ICs yielded faster dissolution and increased local availability for cutaneous absorption (examples include caffeine–HPβCD hydrogels, nanosponge β-CD hydrogels with imiquimod, and HPβCD hydrogels with formononetin) [75,76]. Conversely, excessive complexation (high CD/drug molar ratios or very stable complexes) can reduce the free drug concentration and therefore reduce flux [70,77,78].

Vehicle components and their effect on SC hydration are additional determinants of skin permeability. Increased local hydration typically loosens lipid packing and increases diffusivity within the SC interstices [79]. CDs formulated in hydrating aqueous vehicles or co-solvent systems will therefore combine their intrinsic complexation and any lipid-interactive properties with vehicle-induced changes in SC water content, leading to formulation-dependent outcomes for permeation [69,80].

Experimental reports illustrate the range of CD effects on skin. Argenziano et al. demonstrated that a β-CD nanosponge hydrogel increased skin permeation and the retention of imiquimod in vitro [79]. Dias et al. found that HPβCD-containing hydrogels increased the dissolution and skin availability of caffeine and formononetin, with consequent enhancement of epidermal deposition or flux in model systems [75]. Monti et al. intentionally used α-CD complexes to limit the percutaneous absorption of UV filters and retain actives in the outermost layers [81]. Conversely, studies have demonstrated that derivative chemistry and concentration critically dictate whether the net effect is enhanced local delivery, increased systemic absorption, or barrier impairment [82,83].

### 3.2. Effects on Skin Barrier Function

SC is the primary barrier preventing most molecules from entering intact skin [84]. It limits the entry of lipophilic molecules and blocks hydrophilic and large molecules. Factors such as polarity, molecular weight, formulation composition, and solubility in oil and water significantly influence molecular permeation. Typically, for effective skin delivery, a molecular weight of up to 500 Da is considered. Achieving an optimal balance between skin penetration and sufficient retention of active compounds at therapeutic concentrations remains a challenge [60]. Cyclodextrin molecules are relatively large, with molecular weights ranging from about 1000 to over 1500, and have a hydrated outer surface. Under usual conditions, they face significant challenges in skin barrier permeability. It is important to distinguish between topical and transdermal delivery: topical delivery targets a local effect, while transdermal delivery aims for a systemic effect, with the skin serving as the entry point for active ingredients [85]. This distinction is crucial in understanding how CDs contribute to enhancing delivery mechanisms. CDs significantly enhance both topical and transdermal delivery, but being generally too large to penetrate the SC, they act as permeation enhancers by increasing the solubility of active ingredients and creating an in situ reservoir effect [60,86].

SC lipid composition and organization underpin barrier integrity. Experimental studies show that ceramides, cholesterol, and free fatty acids form unique lamellar phases essential for low permeability, and that both composition and morphological organization determine barrier resistance to diffusion and water loss [60,84,87]. Functional metrics such as transepidermal water loss (TEWL) and SC hydration are established surrogate measures of barrier performance and are sensitive to compositional and structural perturbations of the SC lipids [88].

The experimental record identifies the capacity of certain cyclodextrins to extract SC lipids and to interact with lipoprotein surfaces, effects that are expected to decrease lamellar cohesion and increase permeability and water loss [86,89]. Research confirms that perturbations that reduce intercellular lipid content or interfere with lipid delivery to the SC are associated with delayed or impaired barrier function [90,91]. Clinically, reduced ceramide content and altered lipid profile correlate with increased TEWL and skin dryness in various conditions. Conversely, lipid-restorative interventions (topical ceramide/phospholipid preparations) normalize TEWL and hydration and stabilize barrier function [92,93].

Barrier impairment induced by lipid removal or disorganization increases skin susceptibility to irritants and can amplify cutaneous immune responses as keratinocytes and epidermal immune networks sense and react to barrier disruption [94,95]. The experimental literature on CDs emphasizes lipid extraction and percutaneous absorption changes. Formulations that substantially reduce SC lipid content could elevate the risk of inflammation and irritant sensitivity unless mitigated by formulation design or exposure control [94].

The magnitude of barrier modulation depends on CD type (native vs. substitute), substitution pattern (e.g., methylation), concentration, dosing regimen, and the vehicle matrix. Methylated β-CDs tend to be most efficacious as lipid chelators, while hydrophilic derivatives (e.g., HPβCD) have higher aqueous solubility and, in many experimental settings, show less propensity for cholesterol extraction and barrier disruption [82,83,96].

Because CD–skin interactions may be subtle and formulation-dependent, a suite of experimental endpoints is used to assess barrier effects and compatibility: corneoxenometry and colorimetric assays can detect lipid bilayer disruption or protein denaturation of corneocytes [97]. TEWL, skin hydration, pH, and clinical signs are assessed as part of in vivo physiology during repeated application [70,98]. Ex vivo and in vivo permeation models document flux, retention, and systemic exposure [75,80]. Comparative studies emphasize that CD chemistry and delivery matrix determine whether barrier perturbation is negligible (as reported for many HPβCD-based topical systems) or detectable (notably for methylated derivatives or aggressive solvent/vehicle combinations) [70,97].

Time course matters as single applications may produce transient effects (e.g., increased hydration and flux), while repeated or high-dose exposure (particularly with lipid-extracting derivatives) can cause cumulative depletion of key lipid species and longer-lasting barrier impairment [70,99].

Accordingly, assessment of TEWL, lipid composition, and clinical irritation/sensitization endpoints is necessary when CD-containing topical products are developed.

### 3.3. Influence of Cyclodextrin Type

Both the chemical identity of the cyclodextrin and the exposure concentration (and duration) affect the balance between favorable encapsulation effects and undesirable lipid interactions.

The native cyclodextrins (α, β, and γ) differ in cavity size and in their affinity for guest molecules. Moreover, chemical substitutions alter water solubility and surface properties, affecting the propensity of a given CD to interact with lipids. Substitution (hydroxypropylation, methylation, sulfobutylation, etc.) modifies aqueous solubility, lipophilicity, complex stability, and interactions with biological membranes [81,82,100]. Cavity size and substitution, therefore, guide CD selection for a particular active and desired cutaneous outcome (e.g., deeper permeation versus surface retention) [81,82]. Comparative studies show that parent β-cyclodextrin surfaces can engage lipoprotein structures and extract lipids, whereas some more water-soluble derivatives exhibit markedly different (often reduced) lipid interactions. Thus, the selection of CD type is crucial for tuning skin compatibility. Derivatives with lowered affinity for membrane lipids (but retaining adequate complexation for the intended active) are generally preferable when minimization of barrier perturbation is required [69].

HPβCD is widely used in topical and cosmeceutical preparations because it markedly increases the aqueous solubility of poorly soluble actives and, in many formulations, shows acceptable skin compatibility. Several studies of HPβCD hydrogels and emulsions report enhanced epidermal deposition or improved dissolution without overt barrier disruption in routine application models [70,75,80]. In contrast, methylated β-CDs (e.g., RMβCD) are more lipophilic, bind membrane cholesterol more effectively, and consequently exhibit greater permeation-enhancing potency but also higher potential for membrane perturbation and cytotoxicity in some models [69,83].

The extent of lipid extraction and hence barrier perturbation is concentration- and exposure-dependent. Percutaneous absorption studies that compare derivatives and applied doses report differential enhancement effects consistent with a dose–response relationship for lipid binding and for net permeability change [66,69]. Consequently, lower CD concentrations, shorter exposure times, and formulation strategies that sequester CDs within vehicle matrices can limit adverse lipid interactions while preserving beneficial encapsulation properties. At moderate CD concentrations, increased solubilization and dissolved drug availability promote flux, while at high CD concentrations, the fraction of the drug retained in stable ICs can reduce free drug activity and thereby decrease flux. This sequestration effect has been observed experimentally for HPβCD in semi-solid gels and is an important formulation consideration [14,101]. The association constant of the complex, the rate of dissociation at the skin surface, and the presence of competing binding partners in the vehicle and SC all modulate this balance [81,102].

The formulation matrix strongly influences how CD type and concentration translate into skin outcomes. Vehicles that maintain a depot of the complexed drug or that reduce CD availability to the SC (e.g., lipid-rich emulsions) may reduce lipid extraction relative to aqueous or simple co-solvent systems, while hydrating vehicles can increase CD mobility and enhance diffusion through loosened lipid packing [66,79].

The global effect of a given CD on skin permeation is not intrinsic to the CD alone but is emergent from its interactions with the formulation architecture. For instance, CDs embedded in nanosponge hydrogels can provide controlled release and dermal retention, while CD–drug complexes loaded into elastic liposomes or nanoemulsions may favor deeper penetration because the carrier enters follicular or intercellular pathways [80,103,104].

Comparative experimental work confirms these principles: the same active complexed with different CDs and formulated in different matrices shows divergent penetration and retention profiles (e.g., tretinoin/dimethyl β-CD systems, ferulic acid/α-CD, and various HPβCD hydrogel systems), hence systematic screening of the CD type, CD/drug ratio, vehicle, and dose regimen is necessary to achieve the intended cutaneous distribution with acceptable compatibility [75,76,101,102].

Therefore, formulation design must be considered in conjunction with CD chemistry and dose to achieve the desired balance of efficacy and compatibility.

**Figure 5:**
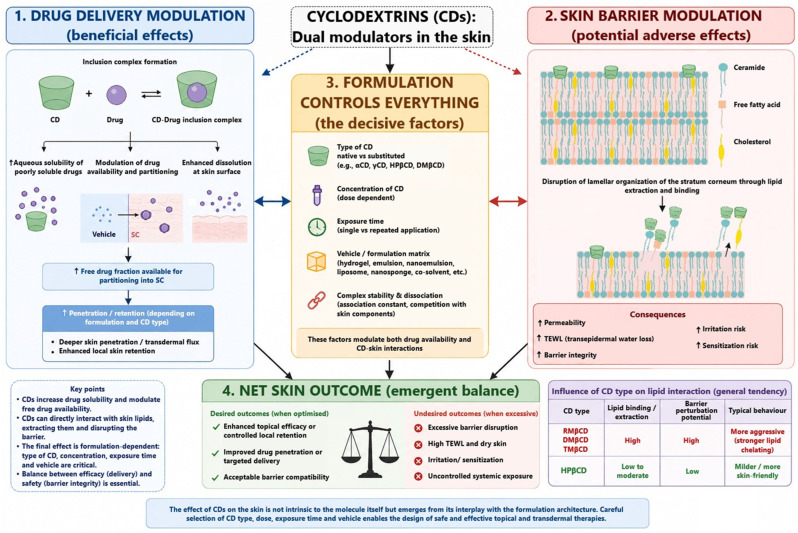
Dual role of cyclodextrins in skin delivery: formulation-dependent modulation of drug availability and skin barrier integrity. Cyclodextrins may improve topical delivery by forming inclusion complexes, increasing the apparent solubility of poorly soluble actives, modulating the free drug fraction, and enhancing drug partitioning and retention in the stratum corneum. At the same time, some CD types, particularly under less favorable formulation conditions, may interact with stratum corneum lipids, disrupt lamellar organization, and increase barrier perturbation. The overall skin outcome depends on CD type, concentration, exposure time, formulation vehicle, and complex stability/dissociation behavior. Created in BioRender. Moaca, E. (2026).

To facilitate the integration of the mechanistic concepts discussed in Section 3.1 and Section 3.2, Figure 5 summarizes the dual role of cyclodextrins in topical delivery. On the one hand, CDs can enhance drug solubility, modulate the free drug fraction, and improve drug partitioning and retention in the skin. On the other hand, depending on CD type, concentration, formulation matrix, exposure time, and complex stability, they may also interact with stratum corneum lipids and perturb barrier organization. The net cutaneous outcome, therefore, reflects a formulation-dependent balance between beneficial delivery modulation and potential barrier-related adverse effects.

## 4. Safety Assessment and Toxicological Evaluation of Cyclodextrins for Skin Application

CDs are used as encapsulating agents to improve the solubility and stability of dermatologic and cosmetic actives, and to modulate their release and local disposition in topical matrices [1,2]. By altering the free/unbound fraction, release rate, and local availability of guest molecules, CDs can influence both the efficacy and local safety profile of the active substance and the formulation as a whole. Thus, a specific, formulation-contextualized safety assessment is essential for skin applications [23,105,106]. CDs are available in native forms (α, β, γ) and various chemically modified derivatives (e.g., hydroxypropyl, sulfobutylether, methyl, and cross-linked polymers/nanosponges), with these structural variations significantly affecting solubility and interaction with biological membranes, influencing their toxicological behavior when applied to the skin [23,105].

### 4.1. Safety Assessment of Cyclodextrins for Topical Use

For topical CD formulations, a safety assessment must follow the standard risk assessment sequence (hazard identification, dose–response assessment, exposure assessment, and risk characterization) adapted to the specifics of CD–guest interactions and topical application [106,107]. Integrated approaches to testing that use in vitro/reconstructed human skin models (for irritation, corrosion, and phototoxicity), in vitro cytotoxicity and mechanistic assays, ex vivo permeation studies, and targeted in vivo or clinical testing (e.g., cumulative irritation and skin patch tests) are recommended, although no single test can fully predict clinical tolerance [106,108]. Standardized in vitro assays commonly used in a CD topical safety battery include reconstructed human epidermis (RHE) viability (OECD-type models), cell viability (MTT/ATP) assays, and in vitro phototoxicity tests, combined with percutaneous absorption assays to estimate systemic exposure where applicable [106,107,108].

In topical systems, the relevant exposure metric is not solely the nominal concentration of the CD or active, but the concentration of free (uncomplexed) active available at the SC and viable epidermis, the kinetics of release from the IC or matrix, and vehicle-dependent partitioning of both the complex and free guest molecules [23,105]. Cross-linked CD polymers (nanosponges) can substantially alter the release profile, influencing local peak concentration and exposure duration. Formulation variables (vehicle polarity, surfactants, pH, occlusion) further modulate percutaneous absorption and local tolerability and must be included in exposure assessments [105,109]. For example, HPβCD is favored in mucoadhesive formulations for its high aqueous solubility and low irritancy profile, showcasing the relevance of derivative selection in safety design [110].

A practical preclinical cascade for a CD-containing topical formulation comprises in vitro cytotoxicity and mechanistic assays, in vitro RHE irritation and standardized phototoxicity protocols, ex vivo skin permeation and mass balance studies to estimate dermal and systemic exposure, and targeted animal studies only if in vitro/ex vivo data indicate risk or if regulatory guidance requires them. Clinical tolerance testing (human patch tests and cumulative irritation) completes the dossier for cosmetic or dermatologic use [106,107,108]. Several published formulation studies demonstrate that HPβCD has shown no evidence of local irritancy in mucoadhesive formulations [110], the inclusion of isotretinoin into CD-based carriers reduced local irritation and photodegradation risk in a drug–C-vesicle dual system intended for acne treatment [111], and azelaic acid complexed with HPβCD in nanovesicles was evaluated for safety with a combination of in vitro and tolerance testing [109]. Nanosponges based on cross-linked CDs allow for release tuning and require addressing aspects such as particle size, residual cross-linkers, and biodegradation in safety testing [105,112].

### 4.2. Toxicological Evaluation

In a general sense, cyclodextrins are reported to be safe for skin application. Natural CDs and HPβCD do not disrupt the SC, which diminishes the possibility of skin irritation. Bochot et al. studied this phenomenon by applying 2 mg of CD dissolved in water or scattered in Vaseline on 1 cm^2^ of skin, with results on healthy volunteers indicating a lack of irritation [113].

The concentration limits of native CDs were established using increasing concentrations. At a concentration of 16 mM, a slight irritation, measuring 0.1 on the Draize scale, which ranges from 0 to 4.0, was observed for β-CD, while no irritation response was observed for α-CD and γ-CD. Upon increasing the concentration up to 80 mM, no skin irritation was observed for γ-CD; however, for α-CD and β-CD, the irritation scores were 0.4 and 0.8, respectively. It was concluded that γ-CD is characterized by a very high level of skin safety [114].

In the particular case of methylated CD derivatives, safety is ensured at low concentrations. Due to the high concentrations (10 to 20%) in aqueous solutions or suspensions, they develop the ability to interfere with SC constituents (cholesterol and triglycerides), with the effect being skin irritation [113]. In order to investigate the effect of chemical xenobiotics, which are different from surfactants, on the human SC and to report the compatibility of CDs currently used in pharmaceutical preparations, corneoxenometry, a predictive bioassay, is usually applied, with results showing a tolerance limit of 5% [98].

In vitro evidence indicates that commonly used hydrophilic CD derivatives, particularly HPβ-CD, exhibit low intrinsic cytotoxicity in epithelial cell assays and do not cause direct irritancy at formulation-relevant concentrations, as documented in pharmaceutical film and vesicle studies [23,109,110]. Photobiochemical studies show that complexation with charged derivatives such as SBEβCD can modify the photoreactivity of guest compounds, thus reducing their phototoxic potential, as demonstrated with a SBEβCD formulation of bufexamac [115]. However, CDs are not inert, and methylated β-CDs and certain derivatives may extract membrane lipids (notably cholesterol) from cellular membranes, potentially resulting in cytotoxicity or altered barrier properties [23]. Cross-linked CD polymers (nanosponges) have unique particulate characteristics and a three-dimensional network that can impact both uptake and local cellular responses; in vitro cytocompatibility testing of nanosponges supports acceptable profiles, but results depend on cross-linker chemistry and the extent of cross-linking [105,112].

Clinical and preclinical experience with topical formulations containing HPβCD and other derivatives generally indicates good local tolerance when the formulation design controls free active concentrations and avoids membrane-disruptive CD derivatives [23,110,111]. Important translational limitations exist with in vitro models. Not all in vitro findings (e.g., modest membrane perturbation by methylated CDs) reflect clinically relevant irritation when used topically, while some formulation interactions may only manifest in long-term human applications (cumulative irritation, sensitization), making clinical patch testing and cumulative irritation studies essential steps in safety evaluation [107,108,116]. Evidence shows that CDs can mitigate adverse outcomes related to guest molecules by decreasing photodegradation and the peak concentration of reactive species at the skin. Complexation strategies have been used to reduce phototoxicity from otherwise problematic active ingredients [111,115,117,118].

Mechanisms by which CDs influence topical toxicology include reduction of the active’s free (unbound) activity through complexation and slow release, modulation of percutaneous absorption and partitioning between formulation and skin, direct interactions with skin lipids and proteins (more frequent with membrane-affine derivatives such as methylated CDs), and particle-related effects and potential irritation from insoluble or colloidal CD polymers (nanosponges) or residual materials [23,105,119]. This understanding should guide the selection of in vitro assays (e.g., membrane integrity and lipid extraction assays) and the design of ex vivo permeation studies that evaluate both free and complexed species in donor and receptor compartments [105,106].

### 4.3. Irritation, Sensitization, and Phototoxicity Considerations

Topical product irritation is often multifactorial, influenced by the irritancy of the active, vehicle composition (surfactants, solvents, and pH), and exposure patterns (frequency and occlusion). CDs can either reduce or exacerbate irritation depending on the context [106,120,121]. Encapsulation by CDs, as well as other carriers, reduces the unbound concentration and subsequent irritant responses in various formulation studies; strategies that utilize CD complexation have been proposed specifically to diminish retinoid-induced irritation and protect unstable actives from degradation that could elevate local irritation risks [111,122,123]. The literature demonstrates reduced irritation when actives are incorporated into CD-based carriers, as noted with isotretinoin and azelaic acid formulations [109,111].

Contact sensitization, which is an immunologically driven response distinct from irritancy, shows low potential with native and hydrophilic CD derivatives when tested correctly. Clinical experience with HPβCD in topical pharmaceutical and cosmetic formulations has not prominently identified CD-driven allergic contact dermatitis [23,110]. However, modified structures (novel substituents or cross-linked networks) could present new epitopes or residual impurities, necessitating sensitization testing (e.g., local lymph node assay or validated in vitro alternatives, followed by human repeated insult patch testing) as part of product-specific safety assessments [105,106,107,124]. Established protocols for evaluating sensitization and cumulative irritation in Phase 1 dermatological studies apply to CD formulations and should be utilized to validate low sensitization risks in humans [116,125].

Phototoxicity and photoallergy are critical considerations for topical actives that absorb solar radiation or form reactive photoproducts, with ketoprofen and other nonsteroidal anti-inflammatory drugs (NSAIDs) being well-documented examples of topical phototoxic/photoallergic risks [118]. CDs can substantially influence phototoxic risk by stabilizing the guest, reducing its free concentration at the skin surface, or altering its photochemical pathways. SBEβ–CD complexation has demonstrated a reduction in the in vitro phototoxic potential of bufexamac, indicating that careful derivative selection can alleviate photoreactivity [115]. However, CDs can also enhance the skin absorption of photoreactive guests in specific vehicles, potentially increasing phototoxic risk if release kinetics favor a rise in free concentration within the viable epidermis. Hence, phototoxicity testing (in vitro phototoxicity assays, ex vivo skin irradiation models, and clinical photopatch/photoallergy trials as necessary) is important for formulations containing photosensitive components [105,115,117,118]. Testing batteries should comprise validated phototoxicity protocols and, when appropriate, clinical photopatch assessments following standardized guidance [106,107].

**Figure 6:**
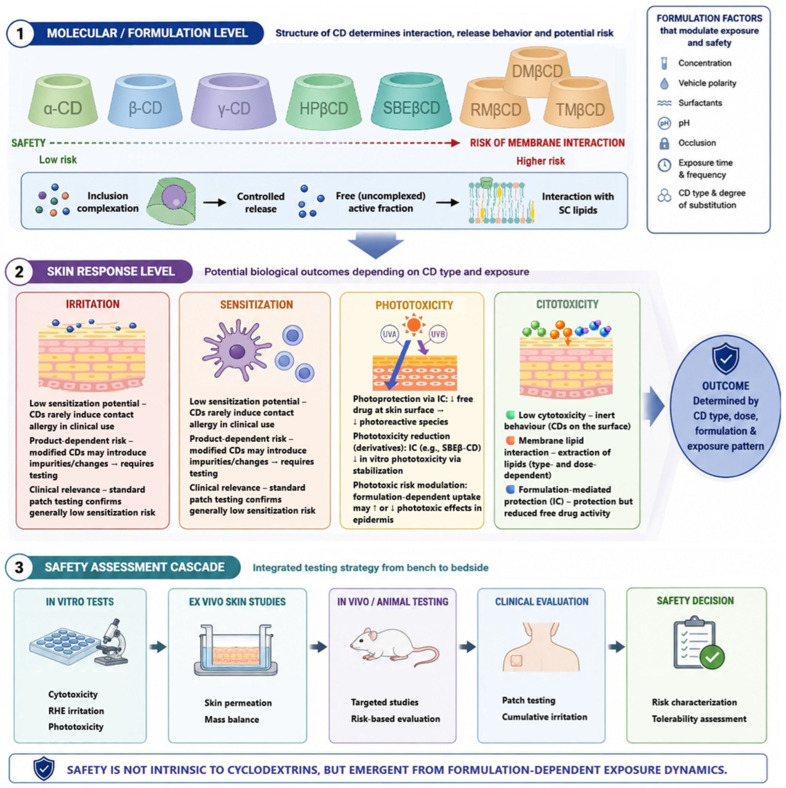
Integrated safety assessment of cyclodextrins for topical applications: from formulation-dependent molecular interactions to skin biological responses, preclinical testing, clinical evaluation, and safety decision-making. The scheme highlights how CD type, degree of substitution, concentration, vehicle characteristics, and exposure conditions influence membrane interaction, irritation, sensitization, phototoxicity/photoallergy potential, and barrier modulation. It also summarizes the safety-assessment cascade from in vitro and ex vivo studies to in vivo and clinical evaluation, culminating in risk characterization, acceptable use-level determination, and regulatory compliance. Created in BioRender. Moaca, E. (2026).

Given that the cutaneous safety of cyclodextrin-based topical systems is not determined solely by the intrinsic properties of the CD molecule, but also by formulation composition and exposure conditions, an integrated view of the safety assessment pathway is useful. Figure 6 summarizes the progression from formulation- and molecular-level determinants to biological skin responses and the stepwise safety evaluation cascade, encompassing in vitro, ex vivo, in vivo, and clinical assessment. The scheme also highlights how these data ultimately support the overall safety decision and inform regulatory compliance for topical products.

## 5. Legislative and Regulatory Aspects

The regulatory relevance of these considerations is closely linked to the integrated safety evaluation cascade summarized in Figure 6.

The regulatory requirements of CDs depend principally on the legal category of the finished product and the claims associated with that product [108,126]. This dual role, utility as an excipient in approved drug products and as an ingredient in cosmetics or consumer dermatological preparations, means that manufacturers, formulators, and safety assessors must consider two distinct regulatory pathways and evidentiary standards when deploying CDs in topical and dermal applications [108]. In jurisdictions with active regulatory reform, such as the United States and the European Union, evolving post-market obligations, animal testing restrictions, and the rise of alternative testing strategies directly affect the pre-market safety dossier for CD-containing dermal products and the means used to substantiate safety and performance [127,128,129].

In the European Union, cosmetics are regulated under a harmonized framework that has, since the early 2010s, explicitly restricted the use of animal testing for cosmetic ingredients and finished products. This regulatory evolution (for example, under the consolidated framework of Regulation (EC) No 1223/2009 and subsequent bans) has driven widespread adoption of in vitro and alternative methods for safety and efficacy substantiation in the cosmetics sector [128,129]. In parallel, the global regulatory landscape has seen a policy push toward non-animal-testing approaches for chemical safety evaluation, influencing how cosmetic dossiers for CD-based formulations are assembled and evaluated [130]. In the United States, cosmetics have been under the FDA’s (Food and Drug Administration) remit since the Federal Food, Drug, and Cosmetic Act, but oversight historically has been less prescriptive than for drugs. Recent statutory modernization (the Modernization of Cosmetics Regulation Act of 2022, MoCRA) has introduced new mandatory obligations, including adverse reaction reporting, enhanced labeling expectations, and formalized safety assessment duties, thereby increasing the regulatory scrutiny of cosmetic products and the ingredients they contain [127,131]. These jurisdictional differences and parallel trends toward enhanced post-market surveillance and alternative testing modalities are an important context for the safety evaluation and regulatory strategy applied to cyclodextrin-containing skin products [127,128].

### 5.1. The European Legislative Framework for Cyclodextrins: Applications in Medicinal Products and Cosmetics

According to Article 65(e) of Directive 2001/83/EC of the European Parliament and of the Council of 6 November 2001 on the Community code relating to medicinal products for human use (OJ L 311, 28 November 2001, p. 67) [131], the European Commission is entrusted with the task of drawing up detailed guidelines. These guidelines should include a list of excipients that must appear on the label of medicinal products and specify how these excipients should be presented [132]. Cyclodextrins (CDs) are not currently included in the guidelines. However, in the current version of the ‘Annex to the European Commission’s Guideline on Excipients in the labeling and package leaflet of medicinal products for human use’ [131], published in 2024, the European Medicines Agency (EMA) has provided warnings on their safety and use in specific contexts, such as oral, nasal, and parenteral administration.

For all routes of administration of cyclodextrin (e.g., Alfadex, Betadex (E 459), γ-CD, SBEβCD, Hydroxypropyl Betadex, and RMβCD), the package insert specifies a threshold of 20 mg/kg/day, accompanied by a recommendation to avoid use in children under 2 years of age unless recommended by a physician. For oral administration at a threshold of 200 mg/kg/day, a warning about potential digestive problems, such as diarrhea, is included. In addition, for parenteral administration exceeding 2 weeks at the same threshold, people with kidney disease are advised to consult a doctor before receiving the drug [131].

At present, the provisions on CDs set out in the ‘Annex to the European Commission’s Guideline on Excipients in the labeling and package leaflet of medicinal products for human use’ are adopted by the national medicines’ regulatory authorities of Ghana, Liberia, Sierra Leone, and Gambia. This initiative is part of a wider effort to harmonize regulatory practices with international standards, promoting the global exchange of medicines and improving public health outcomes [133].

In Regulation (EC) No 1223/2009 of the European Parliament and of the Council of 30 November 2009, on cosmetic products [134], no specific restrictions are imposed on the use of CDs in cosmetic formulations. According to Cosmetics Europe [135], CDs can be used as cosmetic ingredients provided that a qualified safety assessor, acting on behalf of the cosmetic company, evaluates the toxicological profile, the expected exposure, and the purity of the ingredient and considers it safe for the intended use. The Scientific Committee on Consumer Safety provides detailed guidance on how to conduct a safety assessment [136].

In accordance with Article 19 of Regulation (EC) No 1223/2009, the labeling of cosmetic products must include a full list of all ingredients, using the International Nomenclature of Cosmetic Ingredients (INCI) as standard. In addition, a European Commission database, COSING (Cosmetic Ingredients Database) [137], is available to facilitate quick identification of INCI names. By consulting this database, several relevant INCI names can be identified, indicating that these CDs may be used in certain cosmetic products.

### 5.2. The International Regulatory Framework for Cyclodextrins: Global Perspectives and Applications

In Japan, native CDs are listed in The Japanese Pharmacopoeia [138] as natural products and are therefore used with minimal restrictions in both medicines and foods. In Western countries, their ingestion is regulated by the World Health Organization/Food and Agriculture Organization Joint Expert Committee on Food Additives (JECFA) [139,140,141]. Their use as dietary supplements and as pharmaceuticals is approved by the US Food and Drug Administration (FDA) [138]. Native CDs are classified as generally GRAS by the FDA because they are not significantly absorbed upon ingestion. Both α-CD and γ-CD can be consumed without restriction, but oral intake of β-CD is limited to a maximum of 5 mg per kilogram of body weight per day. As for parenteral use of native CDs, this is subject to much stricter regulations [139].

After a comprehensive pre-market safety and efficacy evaluation, the Food Directorate of Health Canada authorized the use of α-cyclodextrin as a food additive. As of 31 May 2023, α-CD has been added to the list of authorized emulsifiers, gelling agents, stabilizers, or thickeners, recognizing its safety and functionality for use in various foods. Thus, α-CD has been approved for use in various food products with specific maximum concentration limits: 1% in beverage whiteners, 3% in icings, unstandardized dips, and unstandardized emulsified sauces (excluding salad dressings), 2% in unstandardized bakery products, 5% in unstandardized salad dressings, and 2.5% in yogurt [142].

The regulatory status of the natural CDs and HPβCD differs across regions. Regarding food approval, all natural cyclodextrins (α-CD, β-CD, and γ-CD) are GRAS in the US and Japan. In Europe, α-CD is listed as a planned food additive. In Europe, β-CD is recognized as a food additive under Commission Regulation (EU) No 1130/2011, while γ-CD is listed as pending for approval. In the United States Pharmacopeia/National Formulary (USP/NF) and European Pharmacopoeia (Ph. Eur.), α-CD, β-CD, and hydroxypropyl-β-cyclodextrin are included, while γ-CD is in the process of being added. In contrast, the Japanese Pharmaceutical Codex (JPC) includes all natural CDs [143].

According to the General Standard for Food Additives (GSFA) provisions for β-CD, the maximum permitted levels vary depending on the food category. β-CD is permitted in chewing gum at a maximum level of 20,000 mg/kg. The maximum level for pre-cooked pasta, noodles, and similar products is 1000 mg/kg. In water-based flavored drinks, including sports, energy, and electrolyte drinks, as well as particulate drinks, β-CD is permitted up to 500 mg/kg. The same maximum level applies to snacks, whether they are potato, potato-based, cereal, flour or starch-based, from roots and tubers, or pulses and legumes [143].

## 6. Cyclodextrins in the Encapsulation of Plant Extracts and Botanical Active Ingredients

**Figure 7:**
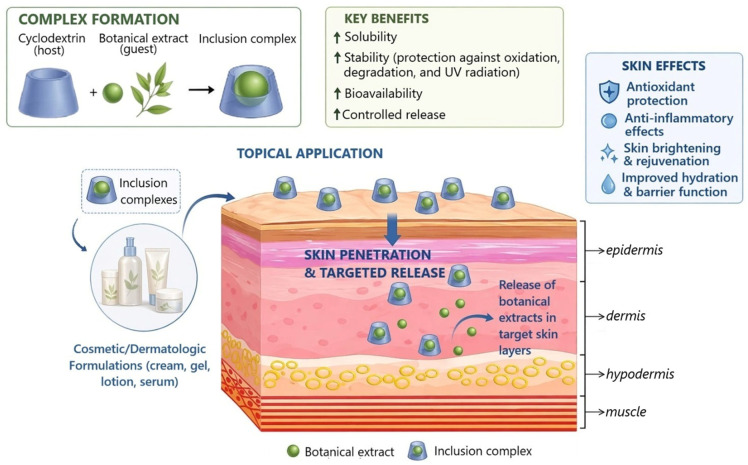
Schematic overview of cyclodextrin–botanical inclusion complexes for skin delivery. Cyclodextrins act as host molecules capable of encapsulating plant-derived guests, thereby improving their solubility, stability, bioavailability, and controlled release. After incorporation into topical cosmetic or dermatologic formulations, such as creams, gels, lotions, or serums, these complexes may facilitate localized delivery of botanical actives to relevant skin layers and contribute to antioxidant, anti-inflammatory, skin-brightening, rejuvenating, hydrating, and barrier-supporting effects. Created in BioRender. Moaca, E. (2026).

Cyclodextrin-based encapsulation provides a useful conceptual framework for understanding how botanical extracts and plant-derived phytochemicals can be transformed into more suitable candidates for topical cosmetic and dermatologic delivery. Through host–guest inclusion complexation, cyclodextrins can accommodate lipophilic or chemically labile constituents within their hydrophobic cavity, while their hydrophilic external surface facilitates dispersion in aqueous or semi-solid formulations. This supramolecular interaction may improve solubility, protect sensitive compounds against oxidation, degradation, and ultraviolet-induced instability, and modulate their release at the skin surface or within selected epidermal and dermal compartments [144,145,146,147,148]. As summarized in Figure 7, these effects are particularly relevant for botanical actives intended to provide antioxidant, anti-inflammatory, skin-brightening, rejuvenating, hydrating, or barrier-supporting benefits.

### 6.1. Rationale for Using Cyclodextrins with Plant-Derived Actives

Plant extracts and isolated phytochemicals constitute a rich and diverse source of functional ingredients for dermatologic and cosmetic applications, combining multiple bioactivities that address key targets in skin care, including antioxidant, anti-inflammatory, anti-aging, wound-healing, and photoprotective effects [144,145,146,147]. Many polyphenols and phenolic acids demonstrate antioxidant and radical-scavenging properties that mitigate ultraviolet (UV)-induced reactive oxygen species (ROS) formation and downstream oxidative damage to lipids, proteins, and DNA. Flavonoids such as quercetin and myricetin, as well as phenolic acids such as ferulic acid, are frequently cited in the dermocosmetic literature for these activities [145,146,147,148,149]. Moreover, some botanical constituents exhibit specific biological actions relevant to skin health, making plant actives attractive multifunctional ingredients for formulations targeting photoprotection, anti-aging, and repair. Common examples include flavonoids that preserve extracellular matrix homeostasis and collagen synthesis after UV exposure, such as baicalein, and triterpenoids with modulatory effects on cell survival and inflammation [148].

Despite their functional appeal, many botanical actives present intrinsic physicochemical and formulation challenges that limit their practical use in topical products. A significant proportion of effective phytochemicals are lipophilic and poorly soluble in water, constraining their incorporation into aqueous vehicles, diminishing bioavailability at the skin surface, and complicating standardization of doses in finished products [81,150]. Additionally, many plant constituents are chemically unstable: various polyphenols and carotenoids are vulnerable to oxidative degradation and thermal or photochemical decomposition during processing and storage, negatively affecting potency and generating undesirable degradation products [150,151]. Essential oils and many terpenes, characterized by volatility and high vapor pressure, can suffer from rapid loss and pose handling difficulties during manufacturing [152]. Furthermore, natural extracts often display batch-to-batch variability in composition, undermining the reproducibility of biological effects and complicating regulatory compliance for topical products [146].

Cyclodextrins provide a versatile strategy to address many constraints associated with botanical actives. Enabling size-selective inclusion of guest molecules makes CDs broadly applicable to various phytochemicals [151]. Formation of non-covalent ICs between a hydrophobic phytochemical (guest) and the CD (host) enhances the apparent aqueous solubility of the guest, allowing a poorly soluble oil or solid to be transformed into a water-dispersible system readily formulated into creams or gels, a phenomenon documented for various plant-derived molecules [153,154]. Additionally, complexation within the CD cavity protects sensitive chemical moieties from external oxidative or photolytic stimuli, enhancing chemical and photostability. Studies have demonstrated improved stability or reduced degradation of photosensitive compounds when complexed with CDs [155].

Beyond improving solubility and protecting chemical stability, CDs can modify several formulation-relevant attributes of botanical actives that are beneficial for dermatologic and cosmetic applications. For instance, cyclodextrin inclusion can reduce the volatility of essential oils and mask undesirable odors, thereby improving product acceptability and the retention of active ingredients in the final formulation [152,156,157]. Encapsulation with CDs also facilitates standardization and handling during manufacturing by producing solid, easily metered powders or stabilizing pigments and oils [157,158]. CD complexation can be precisely engineered to modulate skin permeation and retention. Studies show that the same IC can either enhance delivery to the epidermis or sustain release at the skin surface, depending on the CD type, guest physicochemical properties, and the formulation [146,152,156,157]. CDs can also integrate seamlessly with other carrier approaches—such as liposomes or hydrogels—allowing for dual-carrier strategies that combine stability gains with controlled release [155,156,157].

The selection of native CDs versus chemically modified derivatives helps tailor the system to match guest size, solubility goals, and formulation needs. Modified CDs often exhibit enhanced aqueous solubility and different complexation affinities compared to native counterparts, demonstrating improved dissolution and bioavailability in multiple systems [154,156,157]. The method of complex preparation (e.g., kneading and co-evaporation) can also influence the degree of inclusion and solid-state properties. Certain processes yield amorphous inclusion products with enhanced dissolution characteristics [159]. It is essential to recognize that optimization of complexation parameters is necessary, with studies finding that some preparation methods can result in incomplete inclusion or lower biological activity compared to the free extract, highlighting the need to balance physicochemical stabilization against the preservation of bioactivity [160].

Concrete examples from the literature illustrate the practical benefits of CD encapsulation for plant-derived actives. For instance, baicalein complexed with a cysteinyl β-CD derivative demonstrated superior restoration of collagen synthesis in UV-exposed fibroblasts compared to the free flavonoid, linking enhanced solubility and stability to improved efficacy in anti-aging formulations [151]. Ferulic acid–CD complexes have been developed for topical photoprotection and show altered skin permeation and distribution, affirming the role of CDs in regulating dermal exposure of a commonly used phenolic sunscreen adjunct [81]. Additionally, carotenoids encapsulated with β-CD exhibit improved stability and have potential applications as natural colorants [3]. Furthermore, encapsulation of volatile botanical oils in cyclodextrin cavities has been shown to reduce volatility and protect against oxidation [152,157].

### 6.2. Improvements in the Solubility, Stability, and Bioavailability of Botanical Compounds

The suboptimal aqueous solubility, chemical lability, and limited bioavailability of many plant-derived actives constitute significant barriers to their effective incorporation into dermatologic and cosmetic formulations. CD-based strategies have emerged as a widely applicable means to address these interrelated problems [6,32,146,147,161,162]. The hydrophilic exterior and relatively hydrophobic central cavity of CDs allow the reversible inclusion complexation of suitably sized hydrophobic guest molecules. This interaction alters the apparent aqueous solubility and physicochemical behavior of polyphenols, terpenoids, and other botanical actives [6,29,163]. The consequences of such complexation for topical and cosmetic delivery are threefold and interdependent: enhancement of solubility and dispersion of poorly water-soluble phytochemicals, protection from chemical and photochemical degradation pathways, and modulation of local bioavailability and tissue penetration through increased local thermodynamic activity, prolonged residence time, or controlled release from complexed or polymeric CD systems [6,29,32,163,164].

The mechanistic basis for solubility enhancement by CDs is both thermodynamic and structural: the formation of an IC places the nonpolar moieties of the guest within the CD cavity, while the outer hydroxylated rim enhances water solubility for the entire complex. This results in a shift in the distribution of guest molecules towards a soluble complexed state, thereby increasing apparent solubility and dispersion in aqueous media [6,29,32,146,147,163]. Practical formulation outcomes arise from physical transformations associated with complexation, such as the reduction of guest crystallinity or conversion to an amorphous state, the improved wetting and dispersibility of powders, and the capability of CD derivatives or cross-linked CD networks to form nanoscale carriers (e.g., nanosponges and polymeric CD matrices) that further enhance aqueous dispersion and control release kinetics [165,166,167]. These multiple, partly overlapping mechanisms enable formulation strategies ranging from simple binary ICs (drug + native or substituted CD) to more elaborate solid dispersions, spray-dried powders, and composite nanocarriers that combine CD inclusion with other excipient functionalities [165,168].

Resveratrol is a well-known example of how cyclodextrin complexation can improve the solubility and dispersion of a poorly water-soluble phytochemical. Numerous studies have documented that inclusion into β-CD or HPβCD increases the aqueous dissolution rate and apparent solubility of trans-resveratrol, facilitating formulation into stable aqueous systems, including nanoemulsions, spray-dried powders, and hydrogels [168,169,170]. For example, Escobar-Avello et al. found that the complexation of crude botanical extracts with HPβCD and concurrent spray-drying has yielded high encapsulation efficiencies (for instance, an 80.5 ± 1.1% overall encapsulation efficiency for a grape cane phenolic extract) and converted heterogeneous, sparsely soluble extract fractions into readily dispersible powders for topical or oral applications [167]. Cyclodextrin-based nanosponges and cross-linked CD polymers have similarly improved dispersion: resveratrol-loaded nanosponges produced through β-CD cross-linking exhibited enhanced aqueous availability and superior mucosal accumulation and transdermal permeation in a pigskin model compared to the unformulated drug, suggesting that CD-derived particulate platforms translate inclusion into measurable gains in tissue delivery [165]. Complementary colloidal approaches that incorporate preformed CD–resveratrol ICs within phospholipid-stabilized nanoemulsions or via dual-encapsulation strategies further enhance aqueous dispersion and can be optimized for particle size, loading, and release [169,171].

Protection from chemical and photochemical degradation is another well-documented benefit of CD complexation. The physical shielding of light- and oxygen-sensitive chromophores within the CD cavity, coupled with the reduced molecular mobility of complexed guests and lowered exposure to bulk solvent, decreased susceptibility to oxidation, hydrolysis, and light-induced isomerization or oligomerization in various polyphenolic structures. Specifically, for resveratrol, both covalent conjugation to CD derivatives (such as resveratrol-functionalized carboxymethyl β-CD) and non-covalent ICs have been shown to maintain photostability under UV exposure while preserving antioxidant function compared with uncomplexed resveratrol. The incorporation of CD complexes into solid or semi-solid matrices (including spray-dried powders, hydrogels, and polymer matrices) further stabilizes the active compound in formulated states [172,173]. The practical outcome is that CD complexation can extend the shelf stability of botanical actives, as well as preserve their biological activity after formulation. For instance, some CD polyphenol systems have demonstrated extended in vitro antioxidant performance, with polymeric CD matrices releasing resveratrol over prolonged periods while sustaining free radical scavenging activity for weeks to months [170,174].

The impact of cyclodextrin complexation on bioavailability and skin penetration is context-dependent but mechanistically sound. CDs themselves are hydrophilic and do not readily cross lipophilic biological membranes in their native forms. Consequently, the principal mechanism by which CDs enhance tissue delivery is indirect: complexation increases aqueous solubility and local concentration. The dynamic equilibrium between complexed and free guest molecules results in elevated thermodynamic activity at the vehicle–skin interface, driving partitioning into the SC and deeper layers [6,29,32,146,147,162,163]. Empirical studies corroborate this framework, a good example being that resveratrol formulated as CD-based nanosponges or CD-stabilized nanoemulsions displayed improved permeation across ex vivo pigskin membranes and increased accumulation in mucosal tissues compared to the unformulated drug [165,166,168]. Incorporating resveratrol–HPβCD complexes into topical hydrogels produced formulations amenable to wound care testing and trends toward enhanced fibroblast migration in scratch assays, aligning with improved local delivery and preserved bioactivity post-complexation [166]. In cases where tissue penetration across biological barriers was necessary, the appropriate selection of CD type and carrier design enabled measurable transport. For example, crocetin complexed with γ-CD demonstrated enhanced central nervous system delivery in preclinical models [175]. Overall, evidence indicates that CDs do not serve as membrane carriers but can effectively modulate local bioavailability and penetration when integrated into rational formulations [164,165].

Beyond modulating solubility, stability, and passive penetration, CD complexation is associated with preserved or enhanced biological activity in vitro and within formulation models. Several studies show that CD-complexed resveratrol retains or exhibits an enhanced antioxidant capacity relative to free resveratrol in aqueous dispersions and following incorporation into delivery systems. Additionally, CD-based carriers can potentiate antimicrobial or cytoprotective responses in cell-based or ex vivo assays [168,169,170,176]. For complex botanical extracts, CD encapsulation can selectively enrich soluble fractions and protect labile constituents, resulting in improved functional outcomes as demonstrated by extract-level antioxidant assays and targeted bioassays after formulation [163,167,177,178,179]. These observations highlight a critical point for dermatologic and cosmetic product development: inclusion into CDs can be a valuable tool not only for facilitating physical incorporation but also for preserving and enhancing the functional properties of botanical actives in vitro and under product-relevant conditions [169,170].

Successful translation of the mechanistic benefits of CD complexation into robust formulations requires attention to CD type, guest-dependent complex stability, and the broader excipient environment. The cavity diameter and substitution pattern (e.g., α-, β-, and γ-CD rings, hydroxypropylation, methylation, carboxymethylation) may influence host–guest affinity, thereby affecting the balance between solubilization, protection, and release. For instance, β-CD derivatives are commonly used with polyphenols such as stilbenes and flavonoids, while γ-CD may be preferred for larger carotenoids such as crocetin [55,180,181,182]. Conversely, excessively strong complexation can limit bioavailability by reducing the free fraction, while weak complexation may provide insufficient protection [163,164,183,184,185]. Various formulation techniques, including spray-drying with HPβCD and carriers such as maltodextrin to create free-flowing powders, forming nanosponges or cross-linked CD particles for sustained release, and incorporating ICs into nanoemulsions for combined partitioning pathways, are pragmatic options to tailor solubility, stability, and release depending on the intended dermal application [165,167,169]. Formulation data underscore the breadth of potential outcomes, as spray-dried HPβCD–extract powders achieved high encapsulation efficiencies (≈80% overall) in grape cane studies, while β-CD-based nanosponges improved the permeation and accumulation of resveratrol in pig skin, and polymeric CD matrices provided sustained release with continued in vitro antioxidant activity [174,186]. In addition, CD–resveratrol ICs in ultrasonically prepared nanoemulsions exhibited superior UV stability while preserving radical scavenging capabilities relative to the free drug [169,170,176].

### 6.3. Botanical Extracts Complexed with Cyclodextrins

A broad spectrum of botanical extracts and individual phytochemicals has been successfully complexed with CDs to overcome formulation-limiting properties, such as low aqueous solubility, volatility, and chemical or photochemical lability, as well as limited incorporation into topical carriers. The ultimate goals are to preserve bioactivity (antioxidant, antimicrobial, anti-inflammatory, etc.), improve stability, and facilitate incorporation into semi-solid and polymeric matrices for skin applications [29,32,36,60,157,187]. Process variables such as co-precipitation, spray-drying, paste/slurry complexation, and ultrasonic-assisted methods materially affect encapsulation efficiency and retention. Studies highlight the importance of those variables that are directly relevant to the stability of dermatologic and cosmetic preparations containing botanical actives such as essential oils and phenolic compounds [188,189,190,191].

In line with current analytical recommendations for CD-based systems, particular attention should be given to the way solubility improvement and IC formation are documented. Whenever available, quantitative aqueous solubility or dissolution data and solution-state NMR evidence in D_2_O provide strong support for the formation of water-compatible host–guest systems. However, in the botanical extract studies summarized in Table 3, D_2_O-NMR data were generally not reported, and the available evidence was instead based on complementary techniques such as thermal analysis, chromatographic profiling, microscopy, release testing, permeation/retention studies, or molecular simulations. Therefore, the table reports the solubility-, dispersion-, release-, or skin-delivery-related outcomes explicitly provided by the original studies, without extrapolating unavailable NMR data.

Essential oils (EOs) and volatile terpenoids represent a class of botanical actives for which CD complexation has been particularly intensively explored, as CDs reduce volatility, mask odor, and moderate dermal irritancy while enhancing aqueous dispersibility and thermal stability. Representative examples include the encapsulation of *Lippia gracilis* essential oil in β-CD, where ICs were developed as environmentally safer formulations for larvicidal activity, and where the preparation method affected oil retention and loading [167]. An analogous β-CD inclusion of *Lippia* (*Aloysia citriodora*) essential oil has been reported, with measurable antimicrobial effects after complexation [192]. HPβCD has been used extensively with volatile oils. Studies revealed that ultrasonic-assisted inclusion of lemongrass essential oil in HPβCD produced amorphous non-crystalline ICs with characteristic X-ray diffraction (XRD) shifts and improved retention of volatile components [193]. Additionally, it was found that HPβCD encapsulation of star anise or black pepper essential oils altered component availability and, in some cases, enhanced antibacterial activity, consistent with selective guest inclusion modifying bioactivity profiles [194]. Clove essential oil complexed with HPβCD retained radical scavenging capacity (DPPH assay) and was successfully incorporated into biodegradable chitosan films, demonstrating a pathway from molecular inclusion to filmable topical formats [195]. Encapsulation of cinnamaldehyde in hydroxypropylated β-CD and γ-CDs followed by electrospinning yielded nanofibers with rapid dissolution, enhanced water solubility, improved thermal stability, and retained antibacterial properties, illustrating how CD-based inclusion can be combined with polymer processing for dermal delivery systems [189]. Studies of cinnamon and other EOs emphasize that ICs are a central option among delivery strategies to address volatility, low solubility, and strong odor, reinforcing the translational relevance of these individual reports for cosmetic and topical antimicrobial applications [196,197].

Polyphenolic botanical extracts and isolated flavonoids have likewise been extensively complexed with CDs to address aqueous insolubility and oxidative instability while preserving or enhancing antioxidant efficacy and enabling incorporation into hydrogels, films, or dried powders for topical use. Resveratrol, a stilbene with limited water solubility, has been complexed with HPβCD and incorporated into hydrogels developed for wound treatment, with characterization data supporting inclusion and functional outcomes consistent with improved handling and local bioavailability in topical matrices [166,198]. Phenolic-rich grape cane extract has been encapsulated by HPβCD and maltodextrin by spray-drying, with ATR-FTIR (attenuated total reflectance Fourier-transform infrared spectroscopy) and SEM (scanning electron microscopy) confirming complex formation and with reported maintenance of antioxidant properties, indicating the feasibility of producing dried, easily reconstitutable CD-based ingredients for cosmetics or pharmaceutical semi-solids [167]. Flavonoids such as myricetin and kaempferol derivatives have been subjects of systematic CD screening (β-CD, γ-CD, HPβCD, M-β-CD, and others), demonstrating significant solubility enhancement and pH-dependent binding thermodynamics that inform selection of CD type for topical formulations where the ionization state and microenvironment vary [188,189]. Several contemporary cosmeceutical preparations exploit HPβCD because of its higher aqueous solubility and reduced toxicity, and it has been used effectively to solubilize rutin, kaempferol derivatives, and other flavonoids derived from plants such as *Satureja montana* and related botanicals for antioxidant and wound healing applications [188,199,200].

Terpenoids and photosensitive botanical actives present additional, clinically pertinent opportunities for CD inclusion. Hyperforin, the labile phloroglucinol constituent of St John’s wort (*Hypericum perforatum*) with reparative and anti-inflammatory activities but pronounced sensitivity to photodegradation, has been captured in HPβCD inclusion formulations that increased aqueous solubility and afforded photoprotection while producing measurable functional effects on keratinocyte mechanosensitive Ca^2+^ signaling and ex vivo atopic skin, which were associated with accelerated wound healing. This is good evidence that CD complexation can convert an otherwise impractical topical phytochemical into an actionable dermatologic agent [201]. Psoralens (psoralen and 5-methoxypsoralen) and furanocoumarins used in photochemotherapy and present in *Brosimum gaudichaudii* extracts exhibit very low water solubility. It was observed that complexation with HPβCD improved solubility and favorably modified the solubility–permeability interplay that is important for topical phototherapeutic delivery [202,203]. Curcuminoids and other phenolic pigments have likewise been reported to gain aqueous solubility and enhance photostability upon complexation with β-CD derivatives, facilitating their formulation into light-exposed topical products where photodegradation would otherwise limit utility [166]. Additionally, plant-derived cannabinoids such as delta-8-tetrahydrocannabinol have been shown to benefit from CD complexation with respect to solubility, chemical stability, and permeability in ocular models, illustrating the generalizability of CD strategies to lipophilic plant terpenoids and suggesting analogous opportunities for dermal delivery when regulatory and safety considerations are addressed [204].

The translation of molecular inclusion into practical topical formats has been demonstrated across a range of carrier types. ICs have been incorporated into hydrogels, semi-solid matrices, and wound dressings (resveratrol HPβCD in hydrogels; caffeine HPβCD in self-healing hydrogels), entrapped into biopolymeric films (clove EO-HPβCD in chitosan matrices), processed into spray-dried powders (grape cane phenolics with HPβCD and maltodextrin), and fabricated as electrospun nanofibers (cinnamaldehyde–HPβCD systems). Dual-carrier strategies (cyclodextrin–vesicle hybrids) have combined the benefits of molecular solubilization with enhanced skin targeting and controlled release [77,111,166,167,190,195]. These integration strategies produce measurable formulation advantages, such as increased aqueous incorporation of hydrophobic botanicals, improved thermal and photochemical stability during manufacturing and storage, modified release kinetics compatible with sustained topical exposure, and the ability to produce dried, reconstitutable ingredients for later incorporation into cosmetic or medicated semi-solids [77,111,166,167,190].

Functionally, CD complexation of botanical extracts has yielded repeated reports of enhanced or preserved antioxidant and antimicrobial activity in vitro and improved handling and stability in model formulations. For example, HPβCD inclusion has been associated with increased antibacterial potency for some EOs (a reported four-fold improvement for black pepper oil upon HPβCD encapsulation), while other systems retain or modestly modify radical scavenging capacity (clove EO-HPβCD via DPPH assays; grape cane phenolics in HPβCD retained antioxidant markers post-spray-drying) [167,194,195]. Electrospun cinnamaldehyde–CD nanofibers demonstrated maintained antibacterial activity together with increased thermal stability and rapid dissolution, outcomes that are desirable for dermally applied antimicrobial dressings or cosmeceutical textiles [190]. Selective encapsulation intrinsic to CD chemistry can change the relative availability of volatile components in an EO and thereby alter biological activity profiles, an effect that has been documented in comparative compositional analyses and must be considered when designing skin-directed products [192,194].

A prospective clinical report of a topical Ziyun ointment, a traditional Chinese herbal remedy (from the roots of *Lithospermum erythrorhizon*), formulated as a β-CD complex, indicated improved skin hydration and reduced transepidermal water loss in patients with psoriasis alongside increased cutaneous superoxide dismutase activity, providing direct human evidence that CD-complexed botanical preparations can modulate skin barrier and oxidative status [205]. Ex vivo keratinocyte and atopic skin studies with hyperforin/HPβCD demonstrated enhanced mechanosensitive signaling and accelerated wound healing responses. Resveratrol/HPβCD hydrogels have been advanced specifically for wound care testing, indicating translational potential beyond in vitro assays [166,201].

Despite these favorable reports, several important limitations and practical considerations have emerged from the literature. Selective encapsulation by CDs can alter essential oil composition and, in some cases, slightly reduce oxidation resistance even while improving other stability metrics, so compositional analysis after complexation is essential for activity-directed formulations [29,32,36,60,157,187,194]. The method of complexation (slurry, paste, co-precipitation, spray-drying, or ultrasonic-assisted) materially influences encapsulation efficiency, residual solvent, and inclusion stoichiometry, and therefore affects the downstream performance of a topical product [167,189,190]. Moreover, because CDs are carbohydrate-based, the potential for providing a fermentable substrate to contaminating microorganisms has been raised and should be considered during the development of preservation strategies for topical products [192]. Finally, safety, regulatory acceptance, and local tolerability differ among CD types. HPβCD is widely favored for topical and systemic applications because of improved aqueous solubility and a favorable safety profile, but the choice of derivative must be informed by toxicological, dermal irritation, and regulatory data relevant to the intended route of application [166,187].

Numerous studies have investigated the complexation of plant extracts and isolated phytochemicals with different cyclodextrins in order to improve their suitability for topical use. To provide an overview, Table 3 summarizes some botanical guests that have been formulated with cyclodextrins for possible cosmetic or dermatologic applications.

**Table 3:** Botanical extracts and plant-derived phytochemicals complexed or formulated with CDs for cosmetic and dermatologic applications, indicating the plant/phytochemical guest, CD host, formulation type, reported solubility-, dissolution-, dispersion-, release-, or skin-retention/permeation-related outcomes, and main key findings.

Plant/Phytochemical (Guest)	Cyclodextrin Type (Host)	Formulation	Reported Solubility/Dissolution/Dispersion/Release-Related Outcome	Key Findings	References
*Helichrysum italicum* extract (rich in phenolic acids)	Hydroxypropyl-β-cyclodextrin (HPβCD)	Aqueous solution extraction/direct cosmetic ingredient	HPβCD-assisted extraction produced a water/lactic acid-compatible extract and supported the improved extraction efficiency of phenolic constituents; quantitative aqueous solubility increases of individual compounds were not reported.	High total phenolic content; potent antioxidant activity (DPPH scavenging, reducing power); superior anti-hyaluronidase activity (IC_50_ 14.31 ± 0.29 μL/mL); superior anti-lipoxygenase activity (IC_50_ 0.96 ± 0.11 μL/mL); non-toxic to HaCaT cells up to 62.5 μL/mL; suitable for direct use in cosmetic products.	[206]
*Helichrysum italicum* extract (rich in total phenols and flavonoids)	Hydroxypropyl-β-cyclodextrin (HPβCD)	Aqueous solution extraction/direct cosmetic ingredient	HPβCD-assisted extraction produced a water/lactic acid-compatible extract rich in total phenols and flavonoids; quantitative aqueous solubility increase for individual compounds was not reported.	High total phenol and flavonoid content; potent antioxidant activity; superior anti-hyaluronidase activity (IC_50_ 19.82 ± 1.53 μL/mL); superior anti-lipoxygenase activity (IC_50_ 1.07 ± 0.01 μL/mL); non-toxic to HaCaT cells up to 62.5 μL/mL; suitable for direct use in cosmetic products.	
Propolis polyphenols (organic certified raw propolis from Greek cultivation)	Hydroxypropyl-β-cyclodextrin (HPβCD)	Combinatorial liposome–cyclodextrin system (CRPP) incorporated into an oil-in-water basic cream base (1% CRPP)	Aqueous/propanediol extraction with 5% HPβCD enabled incorporation of propolis polyphenols into a liposome–CD system. Controlled release of total phenolic compounds was 23.99 ± 1.18% at 8 h and 26.04 ± 2.54% at 48 h; quantitative aqueous solubility increase was not reported.	System exhibited significant polyphenol encapsulation efficiency, physicochemical stability, and a controlled release rate (cumulative release of total phenolic compounds was 26.04 ± 2.54% at 48 h). CRPP protected HaCaT cells against the mutagenic effects of UV radiation (single-cell electrophoresis) and lowered UV-induced protein carbonyl content. It also protected reconstituted human skin tissues from UVB-induced lesions and decreased UVB-induced expression of matrix metalloproteinases (a marker of photoaging) at mRNA and protein levels.	[207]
Curcumin (*Curcuma longa*)	Curcumin–β-cyclodextrin complex	Oil-in-water (O/W) emulsions	Cur–βCD emulsions showed improved formulation dispersion/stability compared with Cur powder emulsions, with pH and viscosity stability for up to 90 days and no Cur powder-like agglomeration; quantitative aqueous solubility increase was not reported.	The O/W emulsions containing the curcumin–β-cyclodextrin complex presented great viscosity and pH stability for up to 90 days of storage, contrary to the emulsion with curcumin powder. This emulsion also exhibited the highest antioxidant activity. The complex was implemented to improve the bioavailability, solubility, and stability of curcumin powder.	[208]
Isoflavone formononetin	Hydroxypropyl-β-cyclodextrin (HPβCD)	HPMC hydrogel (physical mixture, without complexation)	No IC was prepared; quantitative solubility enhancement was not reported. HPβCD increased formononetin retention in epidermis + dermis from 0.75 to 1.6 μg/cm^2^, corresponding to an approximately 2-fold increase.	Significantly increased formononetin skin permeation and retention, denoting a skin promoter effect. The concentration of formononetin retained in the dermis plus epidermis was 1.6 μg/cm^2^ with HPβCD, compared to 0.75 μg/cm^2^ without cyclodextrin. HPβCD increased the amount of permeation/retention of formononetin by 2-fold, suggesting potential for anti-aging products. The distribution order in skin layers changed from dermis > SC > epidermis (without CD) to dermis > epidermis > SC (with CD).	[75]
	Methyl-β-cyclodextrin (MβCD)	HPMC hydrogel (physical mixture, without complexation)	No IC was prepared; quantitative solubility enhancement was not reported. MβCD acted as a permeation promoter and reduced SC retention, indicating enhanced passage toward deeper skin layers.	Showed a significant skin promoter effect on formononetin permeation in the absence of biochanin A. The reduction in its retention in the SC when cyclodextrins were present reveals their significant promoter effect on formononetin skin permeation.	
Isoflavone biochanin A	Hydroxypropyl-β-cyclodextrin (HPβCD)	HPMC hydrogel (physical mixture, without complexation)	No IC was prepared; quantitative solubility enhancement was not reported. HPβCD increased biochanin A retention in epidermis + dermis from 2.0 to 2.5 μg/cm^2^, corresponding to an approximately 1.25-fold increase.	Reduced biochanin A concentration in the SC and significantly improved its epidermis and dermis retention. HPβCD increased the amount of permeation/retention of biochanin A by 1.25-fold. The retention in dermis plus epidermis increased from 2.0 μg/cm^2^ (without CD) to 2.5 μg/cm^2^ (with CD). The formulations presented a skin distribution order of dermis > epidermis > SC. Biochanin A showed 2.7 times the permeation capacity in the epidermis and dermis, mainly after incorporation of cyclodextrins.	
Isoflavone biochanin A	Methyl-β-cyclodextrin (MβCD)	HPMC hydrogel (physical mixture, without complexation)	No IC was prepared; quantitative solubility enhancement was not reported. CD incorporation improved epidermis/dermis retention; biochanin A showed a 2.7-fold permeation capacity in the epidermis and dermis, mainly after CD incorporation.	Reduced biochanin A concentration in the SC and significantly improved its epidermis and dermis retention. The formulations presented a skin distribution order of dermis > epidermis > SC. Biochanin A showed 2.7 times the permeation capacity in the epidermis and dermis, mainly after incorporation of cyclodextrins.	
Isoflavones formononetin + biochanin A	Hydroxypropyl-β-cyclodextrin (HPβCD)	HPMC hydrogel (physical mixture, without complexation)	No IC was prepared; quantitative solubility enhancement was not reported. Co-incorporation reduced formononetin permeation even in the presence of HPβCD, whereas biochanin A permeation was not substantially influenced by formononetin.	Formononetin permeation decreased when incorporated together with biochanin A, even with the incorporation of cyclodextrins. The skin permeation of biochanin A was not influenced by the presence of formononetin.	
	Methyl-β-cyclodextrin (MβCD)	HPMC hydrogel (physical mixture, without complexation)		Formononetin permeation decreased when incorporated together with biochanin A, even with the incorporation of cyclodextrins. The skin permeation of biochanin A was not influenced by the presence of formononetin.	
Pomegranate peel extract (*Punica granatum*)	Aqueous 60% β-cyclodextrin solution (hydropro-pyl-β-cyclodextrin, HPβCD)	Cosmeceutical facial cream (nanofibers incorporated)	No quantitative aqueous solubility increase was reported. Encapsulation in βCD polymeric matrix nanofibers enabled incorporation into facial cream and yielded the most stable rheological profile; initial complex viscosity was 2520 Pa·s, with negligible storage-related changes.	Resulted in the most stable formulations (pomegranate creams) compared to the basic cream and the tea tree oil cream. Dynamic mechanical analysis showed acceptable rheological characteristics and negligible changes in storage modulus, loss modulus, and complex viscosity due to storage period or storage temperature. The initial viscosity was 2520 Pa·s.	[209]
Tea tree oil extract (*Melaleuca alternifolia*)	Aqueous 60% β-cyclodextrin solution (hydropro-pyl-β-cyclodextrin, HPβCD)	Cosmeceutical facial cream (nanofibers incorporated)	No quantitative aqueous solubility increase was reported. Encapsulation in βCD polymeric matrix nanofibers enabled incorporation into facial cream with acceptable rheological characteristics; initial complex viscosity was 3720 Pa·s, although viscosity changed under storage.	The addition of the extracts’ nanofibers resulted in products with acceptable rheological characteristics. However, tea tree oil creams presented alterations in complex viscosity under all storage conditions, indicating less stability compared to the pomegranate extract cream. The initial viscosity was 3720 Pa·s.	
*Celastrus paniculatus* seed oil (CPSO)—oleic acid (main fatty acid)	2-hydropropyl-β-cyclodextrin (2-HPβCD)	Serum formulation (also tested as a solid-state complex, dispersion, and gel)	The CPSO:HPβCD 1:3 IC showed total recovery of 87.5 ± 3.4% and improved removability/dispersibility after skin application compared with the oily solution; quantitative aqueous solubility increase was not reported. Chemical stability was improved, with 94.97 ± 1.71%, 90.54 ± 3.41%, and 88.47 ± 4.14% oleic acid remaining in the solid IC after 3 months at 4 °C, 25 °C, and 45 °C, respectively. The serum delivered 32.75 ± 1.25 μg/cm^2^ oleic acid into whole skin after 6 h.	Inclusion complex improved stability (over 80% oleic acid remaining after 3 months) and skin penetration of oleic acid; the serum formulation exhibited the highest cumulative amount of oleic acid in the whole skin (32.75 ± 1.25 μg/cm^2^ after 6 h), had proper viscosity, and was easier to remove by water after skin application compared to the oily solution.	[160]
Onion peel (*Allium cepa*) extract—quercetin and resveratrol	β-cyclodextrin (β-CD)	O/W emulsion (sunscreen formulation)	Encapsulation efficiency was 91.8%. Controlled-release behavior was indirectly suggested by the lower immediate SPF of microencapsulated OP compared with free OP extract at the same concentration (36.11 vs. 60.24 at 0.095 mg/mL) and by superior SPF maintenance in O/W emulsions over 5 weeks; quantitative aqueous solubility increase was not reported.	Microencapsulation ensured the protection of the extract’s photoprotective capacity for longer periods, increasing the sunscreen’s shelf life. The formulation containing the microencapsulated extract presented a higher protection factor over time compared to the formulation containing the free extract. Microparticles displayed superior ability in maintaining sun protection factor (SPF) values over five weeks. Encapsulation efficiency was 91.8%.	[210]
Chamomile (*Matricaria chamomilla*) extract (flower)—antioxidants (rutin/chrysin)	β-cyclodextrin (β-CD)	β-CD complex (co-crystallization method)	No direct aqueous solubility increase was reported. βCD complexes were obtained by co-crystallization with recovery yields of 55–76%; controlled release of estimated antioxidants in 60% and 96% ethanol followed Case II transport.	Complexation achieved via co-crystallization with good recovery yields (55–76%). The thermal stability of the complex is similar to that of the β-CD hydrate, but the hydration water content is lower, confirming IC formation. Controlled release from the complex itself revealed Case II transport mechanisms in 60% and 96% ethanol.	[178]
Chamomile (*Matricaria chamomilla*) extract (leaf)—antioxidants (rutin/chrysin)	β-cyclodextrin (β-CD)	β-CD complex (co-crystallization method)	No direct aqueous solubility increase was reported. βCD complexes were obtained by co-crystallization with recovery yields of 55–76%; controlled release of estimated antioxidants in 60% and 96% ethanol followed non-Fickian diffusion.	Complexation achieved with good recovery yields (55–76%). Controlled release from the complex itself indicated non-Fickian diffusion in 60% and 96% ethanol.	
Chamomile (*Matricaria chamomilla*) extract (all parts)—antioxidants (rutin/chrysin)	β-cyclodextrin (β-CD)	Transdermal pharmaceutical formulation (cream: 10% lanolin, 90% Vaseline)	No direct aqueous solubility increase was reported. Transdermal formulations based on βCD/M. *Chamomilla* extract complexes showed mainly non-Fickian antioxidant release, indicating controlled release from the topical matrix.	Almost all formulations based on β-CD/*M. chamomilla* extract complexes showed non-Fickian diffusion for antioxidant release. Release mechanism difference suggests that H-bonding drives diffusion in the β-CD matrix, while hydrophobic interactions govern controlled release in the transdermal formulation matrix.	
*Silybum marianum* extract (seed)—antioxidants (silibinin/silymarin)	β-cyclodextrin (β-CD)	β-CD complex (co-crystallization method)	No direct aqueous solubility increase was reported. βCD/S. *Marianum* extract complexes were obtained with good recovery yields, and antioxidant release from the complex followed non-Fickian diffusion.	Complexation achieved with good recovery yields (55–76%). Controlled release from the complex itself revealed non-Fickian diffusion.	
*Silybum marianum* extract (seed)—antioxidants (silibinin/silymarin)	β-cyclodextrin (β-CD)	Transdermal pharmaceutical formulation (cream: 10% lanolin, 90% Vaseline)	No direct aqueous solubility increase was reported. Transdermal formulations based on βCD/S. *Marianum* extract complexes showed non-Fickian antioxidant release from the topical matrix.	All formulations based on β-CD/*S. marianum* extract complexes showed non-Fickian diffusion for antioxidant release. Controlled release in the transdermal matrix is mainly attributed to hydrophobic interactions.	
*Silybum marianum* extract (seed)—antioxidants (silibinin)	β-cyclodextrin (β-CD)	Transdermal pharmaceutical formulation (cream: 10% lanolin, 90% Vaseline)	No direct aqueous solubility increase was reported. βCD/silibinin or βCD/silymarin complexes showed higher recovery yields, approximately 87%, than crude extract complexes and non-Fickian release from the formulation.	Complexes had higher recovery yields (87%) compared to crude extract complexes. Release from the transdermal formulation revealed non-Fickian diffusion.	
*Lycium ruthenicum* anthocyanins, specifically petunidin-3-O-(trans-p-coumaroyl)-rutinoside-5-O-glucoside and total anthocyanins	β-cyclodextrin (β-CD)	Aqueous solution extraction	βCD aqueous extraction increased anthocyanin extraction yield compared with pure water and tested hydroalcoholic solvents. Optimized extraction used 1.65% βCD at 50 °C for 30 min with a liquid/solid ratio of 15:1; quantitative aqueous solubility increase of isolated anthocyanins was not reported.	β-CD solution yielded a higher extraction of *L. ruthenicum* anthocyanins compared to water, 50% ethanol, and 70% ethanol (in terms of extraction yield). Optimal extraction conditions were found using a 1.65% β-CD solution at 50 °C for 30 min.	[211]
*Lycium ruthenicum* anthocyanins, specifically petunidin-3-O-(trans-p-coumaroyl)-rutinoside-5-O-glucoside and total anthocyanins	Hydroxypro-pyl-β-cyclodextrin (HPβCD)	Aqueous solution extraction	Aqueous HPβCD improved extraction compared with water for the main petunidin derivative, but βCD solutions produced higher extraction yields than aqueous HPβCD; quantitative aqueous solubility increase was not reported.	β-CD solutions produced higher extraction yields than aqueous HPβCD solutions. The β-CD solution resulted in a higher extraction yield than the 1% HPβCD solution (though the difference was not statistically significant compared to 50% methanol and 70% methanol).	
Resveratrol	α-CD	Lipid bilayer (simulating SC interface)	No experimental solubility/dissolution data were reported. MD simulations indicated lower RES release efficiency for αCD/RES compared with βCD/RES at the lipid membrane interface.	β-CD/resveratrol exhibited superior release efficiency at the lipid membrane surface compared to α-CD/resveratrol.	[212]
	β-CD	Lipid bilayer (simulating SC interface)	No experimental solubility/dissolution data were reported. MD simulations indicated that βCD/RES had greater stability than γCD/RES and superior release efficiency compared with αCD/RES; the M-form released RES more efficiently than the D-form.	β-CD/resveratrol IC exhibited greater stability and superior release efficiency at the lipid membrane surface compared to α-CD/resveratrol and γ-CD/resveratrol. M-form (mono-hydroxyl group toward the primary rim) structures facilitated resveratrol release more efficiently than D-form configurations. Poorly stabilized β-CD/resveratrol (1:2) complexes enable easy release of the first resveratrol molecule.	
	γ-CD	Lipid bilayer (simulating SC interface)	No experimental solubility/dissolution data were reported. MD simulations indicated that γCD/RES was less stable than βCD/RES at the lipid membrane interface.	β-CD/resveratrol exhibited greater stability than γ-CD/resveratrol.	
	Hydroxypro-pyl-β-cyclodextrin (HPβCD)	Lipid bilayer (simulating SC interface)	No experimental solubility/dissolution data were reported. MD simulations indicated that substituted βCDs more effectively protected RES; HPβCD/RES showed high binding energy and lower predicted lipid bilayer irritation potential.	It could lessen the irritation to the lipid bilayer. The release of resveratrol is caused by strong intramolecular van der Waals interactions, which help HPβCD exhibit minimal irritation to the skin and is more easily metabolized by the skin.	
Curcumin and Resveratrol (co-delivery)	Cyclodextrin nanosponge	Hydrogel (transdermal delivery system)	Not directly evaluated experimentally in [212]. Prior transdermal nanosponge/hydrogel work cited therein reported improved permeability and stability of curcumin/resveratrol; quantitative aqueous solubility increase was not reported in the source available here.	Significantly improved the permeability and stability of curcumin and resveratrol.	

Note: NR, not reported in the cited study. For complex botanical extracts, quantitative aqueous solubility enhancement is not always reported for each individual constituent; therefore, solubility-related information may refer to improved extraction yield, dissolution behavior, dispersion stability, encapsulation efficiency, release performance, or skin retention/permeation outcomes, depending on the experimental design of the original study. In the studies summarized in this table, D_2_O-NMR evidence was not reported for the listed botanical systems; IC formation or formulation performance was instead supported by complementary methods such as DSC, KFT, FTIR, SEM, HPLC-DAD, HPLC-MS, release studies, permeation experiments, or molecular dynamics simulations.

The comparative analysis summarized in Table 3 also highlights an important methodological limitation of the current literature: similar CD–bioactive systems are often investigated using different experimental designs, different solubility or release endpoints, and non-uniform characterization approaches. This heterogeneity complicates direct comparison between studies and may partly explain discrepancies reported for similar guest molecules. Therefore, future studies on CD-based botanical systems intended for dermatologic or cosmetic applications should report a minimum set of standardized parameters, including quantitative solubility or dissolution enhancement, validated analytical accuracy, and direct evidence of IC formation whenever experimentally feasible. In this context, solution-state NMR in D_2_O, particularly ^1^H-NMR combined with DOSY and ROESY/NOESY experiments, is especially valuable because it can support aqueous compatibility, host–guest proximity, stoichiometry, and guest orientation within the CD cavity [49,50,51]. However, for complex botanical extracts or poorly resolved multicomponent systems, NMR should be interpreted together with phase solubility studies, chromatographic quantification, thermal and spectroscopic characterization, release testing, and skin permeation/retention assays. Recommended minimum reporting criteria are summarized in Table 4.

Such reporting criteria would help distinguish true IC formation and CD-mediated solubilization from simple physical incorporation or formulation stabilization. They would also make it easier to compare similar studies performed on the same guest molecule, particularly when different CD derivatives, preparation methods, or topical vehicles are used. For botanical extracts, where multiple constituents may contribute to the final biological effect, these criteria should be applied at least to the main marker compounds or to the most relevant quantified phytochemical classes.

**Figure 8:**
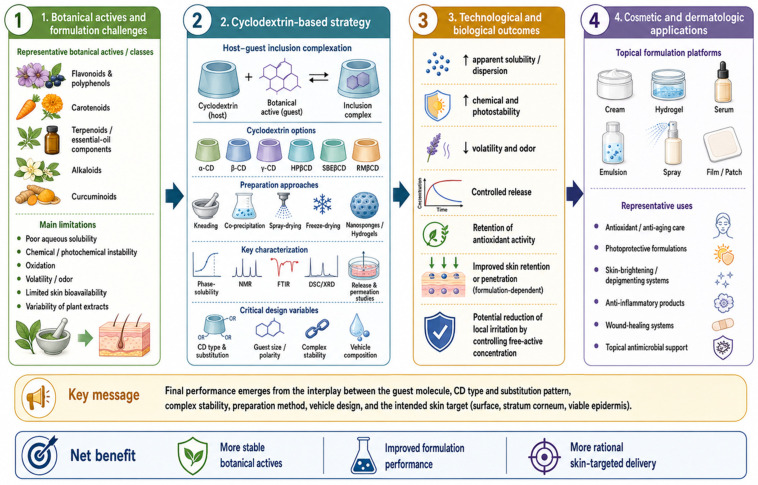
Cyclodextrin-assisted delivery of botanical actives in skin applications: from phytochemical challenges to formulation outcomes. Plant-derived compounds and extracts often present relevant formulation challenges, including poor aqueous solubility, chemical or photochemical instability, oxidation, volatility, or undesirable odor, limited skin bioavailability, and compositional variability. Cyclodextrin-based strategies, centered on host–guest inclusion complex formation and supported by appropriate CD selection, preparation methods, and physicochemical characterization, may improve apparent solubility and dispersion, enhance chemical and photostability, reduce volatility, enable controlled release, preserve antioxidant activity, and modulate skin retention or penetration in a formulation-dependent manner. These advantages support the development of cosmetic and dermatologic topical platforms such as creams, hydrogels, serums, emulsions, sprays, and films/patches for antioxidant, photoprotective, skin-brightening, anti-inflammatory, wound-healing, and topical antimicrobial applications. Created in BioRender. Moaca, E. (2026).

To improve the readability of Section 6 and to integrate the discussion developed across Section 6.1, Section 6.2 and Section 6.3 with the formulation-oriented perspective of Section 6.4, Figure 8 provides a schematic overview of the cyclodextrin-assisted development pathway for botanical actives intended for skin applications. The scheme links the principal physicochemical and technological limitations of plant-derived compounds with CD-based inclusion strategies, key formulation and characterization approaches, and the main technological and biological outcomes that support their cosmetic and dermatologic use. This integrative representation emphasizes that the final performance of CD–botanical systems depends not only on the phytochemical class, but also on CD type and substitution pattern, complex stability, preparation method, vehicle composition, and the intended skin target.

### 6.4. Applications in Cosmetic and Dermatologic Formulations

As summarized in Figure 8, the transition from phytochemical complexation to effective topical application is governed by the interplay between the botanical guest, CD type, preparation method, vehicle design, and the desired cutaneous target.

Cyclodextrins have been extensively utilized to enhance the solubility, stability, and skin penetration of various plant-derived phytochemicals and botanical extracts for topical cosmetic and dermatologic applications.

The practical relevance of cyclodextrin complexation involving botanical actives has been demonstrated in a variety of topical products, ranging from sprays and hydrogels to water-in-oil (W/O) emulsions designed for wound care and anti-aging therapy. In these systems, cyclodextrins act not only as solubilizing and stabilizing agents but also as modulators of dermal penetration, allowing plant phytochemicals to be delivered to the viable skin layers while maintaining acceptable cosmetic properties of the vehicle [213,214,215,216,217,218,219,220,221].

Sawatdee et al. [213] studied *Centella asiatica*, a traditional wound-healing herb, and its ursane-type triterpenes (asiatic acid, madecassic acid, and asiaticoside, madecassoside), which were complexed with HPβCD and formulated into a topical spray solution. The methanolic extract was first assayed for triterpene content and then incorporated into HPβCD. The resulting complex was used to prepare clear yellowish spray formulations (pH 5.5–6.0) containing Eudragit E100, PEG 400, copovidone, glycerol, ethanol, and water. Physicochemical evaluation showed appropriate viscosity, a low contact angle (<20°), and triterpene content close to 100% of the original extract. In a rat full-thickness excision wound model, daily application of the *Centella asiatica*–HPβCD spray (five puffs per wound) produced complete wound closure within 14 days and was non-irritating in a skin tolerance study, supporting its suitability as a cyclodextrin-based phytopharmaceutical for the management of acute skin injuries [213].

Guidi et al. [214] explored *Cenostigma pluviosum* var. *peltophoroides* (syn. *Poincianella pluviosa*), a Fabaceae tree used in Brazil for treating skin lesions, which has also been formulated as a cyclodextrin-based topical system. A crude stembark extract rich in hydrolysable tannins and other polyphenols was complexed with HPβCD in a 1:1 (*w*/*w*) ratio and incorporated into a carbomer hydrogel (gel-CECD). A corresponding gel containing cyclodextrin alone (CD-gel) served as a control. The presence of the complex slightly modified the density and noticeably reduced viscosity compared with the blank gel, but pH remained within the physiological range for skin application. In a rat excisional wound model with paired lesions, treatment with the Cenostigma–HPβCD gel resulted in improved macroscopic healing and histological outcomes relative to the CD-gel, including faster re-epithelialization at days 7 and 14, increased cell proliferation at days 4 and 14, and higher type III collagen deposition at day 10. Photoacoustic spectroscopy further indicated that phytochemicals from the complex permeated into the dermis, confirming effective delivery of the plant extract from the cyclodextrin-based hydrogel [214].

Milk thistle (*Silybum marianum*) provides another example in which cyclodextrin complexation has been exploited to optimize the dermal delivery of a botanically derived antioxidant. Spada et al. [215] prepared a phytocomplex comprising milk thistle dry extract (containing the flavonolignan mixture silymarin) and HPβCD in a 1:4 (*w*/*w*) ratio and incorporated it into two W/O emulsions composed of glycerin, cocoa butter, almond oil, and other lipids. Parallel emulsions containing the non-complexed extract were also produced. In vitro assays showed that complexation modulated the apparent antiradical activity of silymarin but increased the stability of flavonoids over time. In vitro and ex vivo skin-penetration experiments demonstrated that the phytocomplex significantly influenced the percutaneous transport of the extract: HPβCD complexes controlled the permeation profile and enhanced the amount of milk thistle constituents retained in the SC. In vivo tape-stripping studies on human volunteers confirmed that approximately 80% of the complexed extract was absorbed into the SC within 1 h, compared with about 30% for the non-complexed extract, indicating more efficient and targeted deposition in the superficial skin layers [215].

The benefits of optimizing the topical delivery of milk thistle constituents are supported by independent clinical and mechanistic data obtained with non-cyclodextrin formulations. A W/O cream containing 4% *S. marianum* extract, applied for 12 weeks to the cheeks of human volunteers, significantly increased skin hydration, reduced transepidermal water loss, and improved several surface evaluations of living skin parameters compared with the placebo base, indicating measurable anti-aging effects in vivo [216]. Moreover, silymarin itself has been shown to inhibit dermal gelatinolytic activity mediated by matrix metalloproteinase-2 (MMP-2) and matrix metalloproteinase-9 (MMP-9) and to reduce cutaneous inflammation in a mouse ear edema model when applied topically as an ethanolic extract, with up to 74% reductions in edema and the attenuation of histological lesions [217]. These findings suggest that the improved SC targeting and flavonoid stability provided by HPβCD ICs may further enhance the cosmetic and dermatologic performance of silymarin-containing emulsions designed for photoprotection, anti-inflammatory care, and anti-aging indications.

Beyond these specific case studies, cyclodextrin-based systems have been increasingly explored for other plant-derived actives with relevance for skin repair. Studies highlight several examples, including an IC between the citrus flavanone naringin and β-CD that accelerated wound closure in vivo, a coumarin/β-CD complex that improved re-epithelialization and collagen deposition, and a β-CD/polyvinylpyrrolidone-stabilized nanocrystal gel delivering the phytochemicals rutin and thymoquinone, which enhanced skin repair in animal models [218,219,220]. Although these formulations were primarily developed for wound healing indications, the underlying mechanisms, such as enhanced solubility and stability of polyphenols, modulation of their dermal retention, and reduction of local irritation, are equally relevant for cosmetic products targeting barrier restoration, anti-inflammatory care, and scar management.

Plant-derived small molecules used as cosmetic actives can also benefit from cyclodextrin complexation at the preformulation stage. Arbutin, a phenolic glycoside widely used as a skin-lightening agent due to its ability to inhibit melanin biosynthesis, exhibits limited aqueous solubility and poor stability in water, which restricts its incorporation into aqueous or hydroalcoholic topical vehicles. Li et al. [221] prepared an IC between arbutin and HPβCD by freeze-drying a 1:1 molar mixture and demonstrated, using UV spectroscopy, FT-IR, SEM, X-ray diffraction, and thermal analysis, that arbutin was molecularly dispersed in an amorphous state within the cyclodextrin matrix. Complex formation markedly improved the physical and chemical stability of arbutin, including its heat stability, suggesting that such complexes could facilitate the development of more robust and storage-stable skin-whitening formulations [221].

The study of Belo et al. [222] reported the development of a cream formulation containing green tea extract (*Camellia sinensis*), where the active compound epigallocatechin-3-gallate (EGCG) was complexed with HPβCD. The cyclodextrin complex improved the solubility and stability of EGCG, and the cream formulation enhanced the skin penetration of EGCG compared to a control green tea extract preparation. The authors suggested that the topical application of this EGCG-containing cream could provide beneficial antioxidant and anti-inflammatory effects for the skin. Similarly, a quercetin/HPβCD IC was incorporated into a hydrogel formulation and evaluated for wound healing applications. The cyclodextrin complex improved the aqueous solubility and stability of quercetin. In in vitro studies, the quercetin/HPβCD hydrogel exhibited enhanced antioxidant activity and accelerated wound closure compared to a quercetin solution or a blank hydrogel, though further clinical evidence may be required to fully substantiate these findings [222]. Another study developed a lotion formulation containing *Camellia sinensis* leaf extract, where the extract was complexed with β-CD. The cyclodextrin complex improved the solubility and stability of the *Camellia sinensis* extract, and the lotion formulation demonstrated effective UV protection in in vitro studies [223].

Curcumin, a polyphenol derived from turmeric (*Curcuma longa*), has also been complexed with cyclodextrins for topical delivery. A study reported the development of a curcumin/β-CD IC incorporated into a hydrophilic gel formulation. The cyclodextrin complex improved the solubility and stability of curcumin, and the gel demonstrated enhanced skin permeation and improved anti-inflammatory effects compared to free curcumin in in vitro studies [224].

Taken together, these examples indicate that complexation of plant extracts and botanical phytochemicals with cyclodextrins can be successfully translated into diverse topical formats with demonstrable benefits in terms of dermal delivery and biological performance.

## 7. Conclusions

Cyclodextrins represent a versatile class of supramolecular excipients with considerable relevance for dermatologic and cosmetic sciences. Their unique toroidal architecture, characterized by a hydrophobic inner cavity and a hydrophilic outer surface, enables the formation of ICs with a broad range of poorly water soluble, chemically unstable, volatile, or photolabile active ingredients. This property is particularly valuable in skin-targeted formulations, where the performance of an active compound is strongly influenced by solubility, stability, release behavior, skin compatibility, and its ability to reach the intended epidermal or dermal compartment.

The present review highlights that the functionality of cyclodextrins in topical systems is not limited to solubilization. Depending on their type, degree of substitution, concentration, complexation strength, and formulation environment, cyclodextrins can protect labile compounds from degradation, improve the handling and incorporation of botanical actives, reduce volatility or unpleasant odor, and modulate the local availability of guest molecules at the skin interface. However, their effect on skin delivery is formulation-dependent. Complexation does not automatically translate into enhanced penetration, since overly stable complexes or excessive cyclodextrin concentrations may reduce the fraction of free active available for partitioning into the SC. Therefore, rational formulation design must consider the equilibrium between complexed and uncomplexed actives, the release kinetics of the guest molecule, the vehicle composition, and the intended depth of delivery.

The interaction between cyclodextrins and the skin barrier is similarly complex. In many topical systems, especially those based on hydrophilic derivatives such as HPβCD, cyclodextrins may improve local delivery while maintaining acceptable skin compatibility. Nevertheless, certain derivatives, particularly more membrane-active or lipid-interacting cyclodextrins, may perturb SC lipids at higher concentrations or under repeated exposure conditions. This reinforces the need for product-specific evaluation of irritation, sensitization, phototoxicity, transepidermal water loss, skin hydration, and ex vivo permeation behavior. The safety of a cyclodextrin-containing topical formulation should therefore be assessed not only according to the cyclodextrin itself but also according to the guest molecule, the cyclodextrin-to-guest ratio, the vehicle, the exposure site, and the expected frequency of use.

From a regulatory perspective, cyclodextrins occupy an important but heterogeneous position. Several native and modified cyclodextrins have an established history of use in pharmaceutical, food, and cosmetic-related applications. However, their regulatory status and labeling requirements vary according to jurisdiction, route of administration, and product category. In cosmetic products, cyclodextrins must be evaluated within the general safety assessment framework applicable to all ingredients, whereas medicinal products require a more specific excipient-focused approach. This distinction is particularly important for borderline products and dermocosmetic formulations, where claims, mechanism of action, exposure level, and intended use may influence regulatory classification.

A major focus of this review was the encapsulation of plant extracts and botanical active ingredients, which often present significant formulation challenges due to poor aqueous solubility, chemical instability, photodegradation, volatility, variability in composition, and limited skin bioavailability. Cyclodextrin complexation offers a valuable strategy for improving the technological and biological performance of such actives. Examples involving phenolic acids, flavonoids, polyphenols, essential oils, terpenoids, carotenoids, and complex botanical extracts demonstrate that cyclodextrins can enhance dispersion, stability, antioxidant capacity, controlled release, and, in some cases, skin penetration or retention. These findings support the increasing interest in cyclodextrin-based systems as enabling platforms for botanical ingredients in topical cosmetic and dermatologic formulations.

Overall, cyclodextrins should be regarded as adaptable formulation tools rather than universal penetration enhancers or passive carriers. Their performance depends on a precise match between the host structure, guest molecule, preparation method, and final formulation matrix. Future development of cyclodextrin-based skin delivery systems should prioritize systematic host–guest screening, quantitative reporting of solubility and dissolution enhancement, robust physicochemical characterization, validated analytical accuracy, solution-state NMR in D_2_O whenever feasible, standardized in vitro and ex vivo skin models, clinically relevant safety testing, and long-term formulation stability studies. Greater attention should also be given to scalable, reproducible, and environmentally sustainable production methods, especially for advanced cyclodextrin architectures such as nanosponges, polymeric networks, hydrogels, and framework-based materials.

In conclusion, cyclodextrins provide a scientifically grounded and technologically flexible approach for improving the formulation of dermatologic and cosmetic actives, particularly botanical compounds with challenging physicochemical profiles. Their ability to combine molecular encapsulation, stabilization, controlled release, and skin-compatible delivery makes them highly relevant for the development of next-generation topical products. Nevertheless, their successful use requires careful selection, rigorous safety assessment, and formulation-specific optimization. These foundational aspects establish the basis for further exploration of cyclodextrin-containing cosmetic and dermatologic formulations, as well as for future innovations in targeted, sustainable, and clinically validated skin delivery systems.

## Figures and Tables

**Table 1:** Physicochemical properties and cavity dimensions of natural cyclodextrins (α-, β-, and γ-CD), including the number of glucose units, molecular weight, aqueous solubility, height, and outer/inner cavity diameters [30,37,38].

Natural Cyclodextrins	Glucose Units	Molecular Weight (Da)	Solubility (mg/mL)	Height (nm)	Diameter (nm)	
					Outer	Inner
α-CD	6	972	145	0.78	1.37	0.57
β-CD	7	1135	18.5	0.78	1.53	0.78
γ-CD	8	1297	232	0.78	1.69	0.95

**Table 2:** Physicochemical properties and cavity dimensions of selected cyclodextrin derivatives (hydroxypropyl, methylated, and sulfobutylether CDs), including the number of glucose units, average substitution degree or molar substitution, indicative average molecular weight, aqueous solubility, height, and outer/inner cavity diameters [30,37,38].

Cyclodextrin Derivatives	Glucose Units	Average Substitution Degree/Molar Substitution	Indicative Average Molecular Weight (Da)	Solubility (mg/mL)	Height (nm)	Diameter (nm)	
						Outer	Inner
Hydroxypropyl-α-CD	6	MS ≈ 0.60 per glucopyranose unit; total hydroxypropyl substituents ≈ 3.6 per CD molecule	≈1180	≥600	0.78	1.46	0.47–0.53
Hydroxypropyl-β-CD (HPβCD)	7	MS ≈ 0.60–0.65 per glucopyranose unit; total hydroxypropyl substituents ≈ 4.2–4.6 per CD molecule	≈1400	≥600	0.78	1.54	0.60–0.65
Hydroxypropyl-γ-CD (HPγCD)	8	MS ≈ 0.60 per glucopyranose unit; total hydroxypropyl substituents ≈ 4.8 per CD molecule	≈1576	≥500	0.78	1.75	0.75–0.83
Dimethyl-β-CD (DMβCD)	7	DS = 14 per CD molecule; two methyl groups per glucopyranose unit	≈1331	≥500		1.54	0.60–0.65
Randomly methylated-β-CD (RMβCD)	7	Average DS ≈ 12.6 per CD molecule; approximately 1.8 methyl groups per glucopyranose unit	≈1312	≥500		1.54	0.60–0.65
Sulfobutylether-β-CD (SBEβCD)	7	Average DS ≈ 6.5 per CD molecule; approximately 0.9 sulfobutylether groups per glucopyranose unit	≈2163	≥500		1.54	0.60–0.65

Note: For chemically modified CDs, molecular weight values are average and indicative rather than fixed molecular masses, because they depend on the average degree of substitution (DS), molar substitution (MS), substitution pattern, counterion form, and manufacturer specifications. For hydroxypropylated CDs, MS is commonly used because hydroxypropyl side chains may undergo further substitution. Consequently, the values reported in this table should be interpreted as representative average molecular weights corresponding to the indicated substitution ranges.

**Table 4:** Recommended minimum reporting criteria for cyclodextrin-based botanical systems intended for cosmetic and dermatologic skin applications.

Reporting Domain	Minimum Information to Report	Why Is It Important	References
Guest molecule or botanical extract	Botanical source, plant part, extraction solvent, marker compounds, phytochemical class, and compositional profile when available	Enables comparison between studies and reduces ambiguity caused by variability in plant extracts	[49,50,51]
Cyclodextrin host	CD type, derivative, degree of substitution or molar substitution when relevant, supplier/grade, and host/guest ratio	CD substitution pattern and host/guest ratio strongly influence solubility, stability, release, and safety	
Preparation method	Kneading, co-precipitation, spray-drying, freeze-drying, nanosponges, hydrogels, emulsions, or other methods; solvent system, temperature, time, drying conditions, and yield	Preparation conditions influence IC formation, residual solvent, crystallinity, particle properties, and reproducibility	
Quantitative solubility or dissolution enhancement	Intrinsic solubility of the free guest, apparent solubility of the CD system, fold increase, medium composition, pH, temperature, equilibrium time, and replicate number	Allows direct evaluation of the main formulation benefit of CD complexation	
Phase solubility and binding parameters	Phase solubility diagram type, association/stability constant, stoichiometry, confidence interval or standard deviation, and fitting model	Supports mechanistic interpretation and comparison of host–guest affinity across CD types	
Analytical method validation	Quantification method, calibration range, linearity, LOD/LOQ, precision, accuracy, recovery, matrix effects, and statistical analysis	Improves reliability and addresses variability between similar published studies	
Direct IC confirmation	Whenever feasible, solution-state NMR in D_2_O, including ^1^H-NMR chemical shift changes, DOSY diffusion changes, and ROESY/NOESY cross-peaks	Provides molecular-level evidence of host–guest proximity, aqueous compatibility, stoichiometry, and guest orientation	
Complementary characterization	FTIR, DSC/TGA, XRD, SEM/TEM/DLS, molecular docking, or molecular dynamics, depending on system type	Confirms solid-state changes, morphology, particle size, thermal behavior, and predicted interaction mode	
Release and formulation performance	Release profile, kinetic model, antioxidant/activity retention, photostability, volatility reduction, dispersion stability, and formulation stability over time	Links molecular complexation to product-relevant technological performance	
Skin-relevant evaluation	In vitro/ex vivo permeation or retention, donor and receptor media, skin model, mass balance, free versus complexed active comparison, TEWL or irritation endpoints when relevant	Connects CD complexation with intended topical performance and safety	
Data transparency	Raw or supplementary data for solubility, NMR spectra, chromatograms, release curves, and statistical analysis	Facilitates reproducibility, cross-study comparison, and future meta-analysis	

## Data Availability

There are no additional data to be published.

## Abbreviations

The following abbreviations are used in this manuscript:

APIs	active pharmaceutical ingredients
ATR-FTIR	attenuated total reflectance Fourier-transform infrared spectroscopy
CD	Cyclodextrin
CD-MOFs	cyclodextrin-based metal–organic frameworks
CDs	Cyclodextrins
CGTase	cyclodextrin glycosyltransferase
COSING	Cosmetic Ingredients Database
CRPP	Combinatorial liposome–cyclodextrin system
DMβCD	Dimethyl-β-cyclodextrin
DSC/TGA	differential scanning calorimetry/thermogravimetric analysis
EGCG	epigallocatechin-3-gallate
EMA	European Medicines Agency
FDA	Food and Drug Administration
GSFA	General Standard for Food Additives
HA	hyaluronic acid
HPαCD	hydroxypropyl-α-cyclodextrin
HPβCD	hydroxypropyl-β-cyclodextrin
HPγCD	hydroxypropyl-γ-cyclodextrin
IC	inclusion complex
INCI	The International Nomenclature of Cosmetic Ingredients
IR	infrared
ITC	isothermal titration calorimetry
JECFA	Joint Expert Committee on Food Additives
JPC	Japanese Pharmaceutical Codex
MoCRA	The Modernization of Cosmetics Regulation Act
MMP	matrix metalloproteinase
NMR	nuclear magnetic resonance
NSAIDs	nonsteroidal anti-inflammatory drugs
Ph. Eur.	European Pharmacopoeia
RHE	reconstructed human epidermis
RMβCD	randomly methylated-β-cyclodextrin
ROS	reactive oxygen species
SBEβCD	sulfobutylether-β-cyclodextrin
SC	stratum corneum
SEM	scanning electron microscopy
SPF	Sun Protection Factor
TEWL	transepidermal water loss
TMβCD	heptakis (2,3,6-tri-O-methyl)-β-cyclodextrin
USP/NF	United States Pharmacopeia/National Formulary
UV	Ultraviolet
XRD	X-ray diffraction
